# N-Acetylcysteine Applied to Hydrogels: A Comprehensive Systematic Review

**DOI:** 10.3390/gels12080751

**Published:** 2026-08-21

**Authors:** Ermelinda Silvana Junckes, Pâmela Elise Munzlinger, Carla Dalmolin, Marco Fosca, Marcia Margarete Meier, Julietta V. Rau

**Affiliations:** 1 Department of Chemistry, Santa Catarina State University (UDESC), Paulo Malschitzki 200, Joinville 89219-710, Brazil; ermelindasilvana@gmail.com (E.S.J.);; 2 Istituto di Struttura della Materia, Consiglio Nazionale delle Ricerche (CNR-ISM), Via del Fosso del Cavaliere 100, 00133 Rome, Italy; 3 Department of Analytical, Physical and Colloid Chemistry, Institute of Pharmacy, Sechenov First Moscow State Medical University, Trubetskaya 8, Build. 2, 119048 Moscow, Russia

**Keywords:** N-acetylcysteine, hydrogel, biomaterial, therapeutic bioactivity, polymer, crosslinker

## Abstract

N-acetylcysteine (NAC) is a thiol-containing molecule with antioxidant, anti-inflammatory, antimicrobial, and cytoprotective properties that has increasingly been incorporated into hydrogel-based biomaterials. This systematic review evaluates the strategies used to integrate NAC into hydrogels and examines their effects on material properties, controlled release, biocompatibility, and therapeutic activity. The review was conducted according to the PRISMA guidelines using Scopus, PubMed, Web of Science, and SciFinder to identify English-language articles published between 2000 and 2025. Seventy-three studies met the eligibility criteria of this review. NAC has been employed as a physically loaded therapeutic agent, covalently conjugated polymer modifier, contributor to hydrogel crosslinking, metal-coordination ligand, and compound incorporated into nano- and microparticulate carriers dispersed in hydrogel. These approaches enable the modulation of gelation, swelling, adhesion, degradation, and drug-release kinetics. NAC-containing hydrogels have demonstrated robust antioxidant, antimicrobial, antibiofilm, anti-inflammatory, angiogenic, and tissue-regenerative properties in various in vitro and in vivo models, underscoring their potential for advanced biomaterial applications. Release profiles varied from rapid stimulus-responsive delivery to sustained release over several days, depending on the network architecture and the NAC–matrix interactions. However, comparisons among studies were limited by the heterogeneous formulations, release conditions, biological models, and outcome measures. Standardized physicochemical characterization, NAC stability assessment, dose–response evaluation, and rigorous preclinical validation are required to support the translation of NAC-based hydrogels into biomedical applications. We hope that this review will help scientists and innovation centers understand the potential of the NAC-containing hydrogel biomaterials discussed in this study, as well as the opportunities and demands for additional research in this field.

## 1. Introduction

Hydrogels are materials composed of a three-dimensional polymeric network that is cross-linked and capable of absorbing and retaining water while maintaining their shape and structural integrity [1,2]. Owing to the structure provided by the crosslinking network, hydrogels can hold not only water but also particles, cells, and molecules that can be locally delivered via stimulus responses and matrix degradation [2,3]. Hydrogels are advantageous because of their ability to cover irregular areas and polymerize in situ during minimally invasive procedures. Simultaneously, they can be tailored to prepare scaffolds in specific shapes. In the medical field, they can be used as a support and scaffold for cells, mimicking the tissue environment, as a drug delivery system, and as a protective barrier for wounds [1,3,4,5].

**Figure 1:**
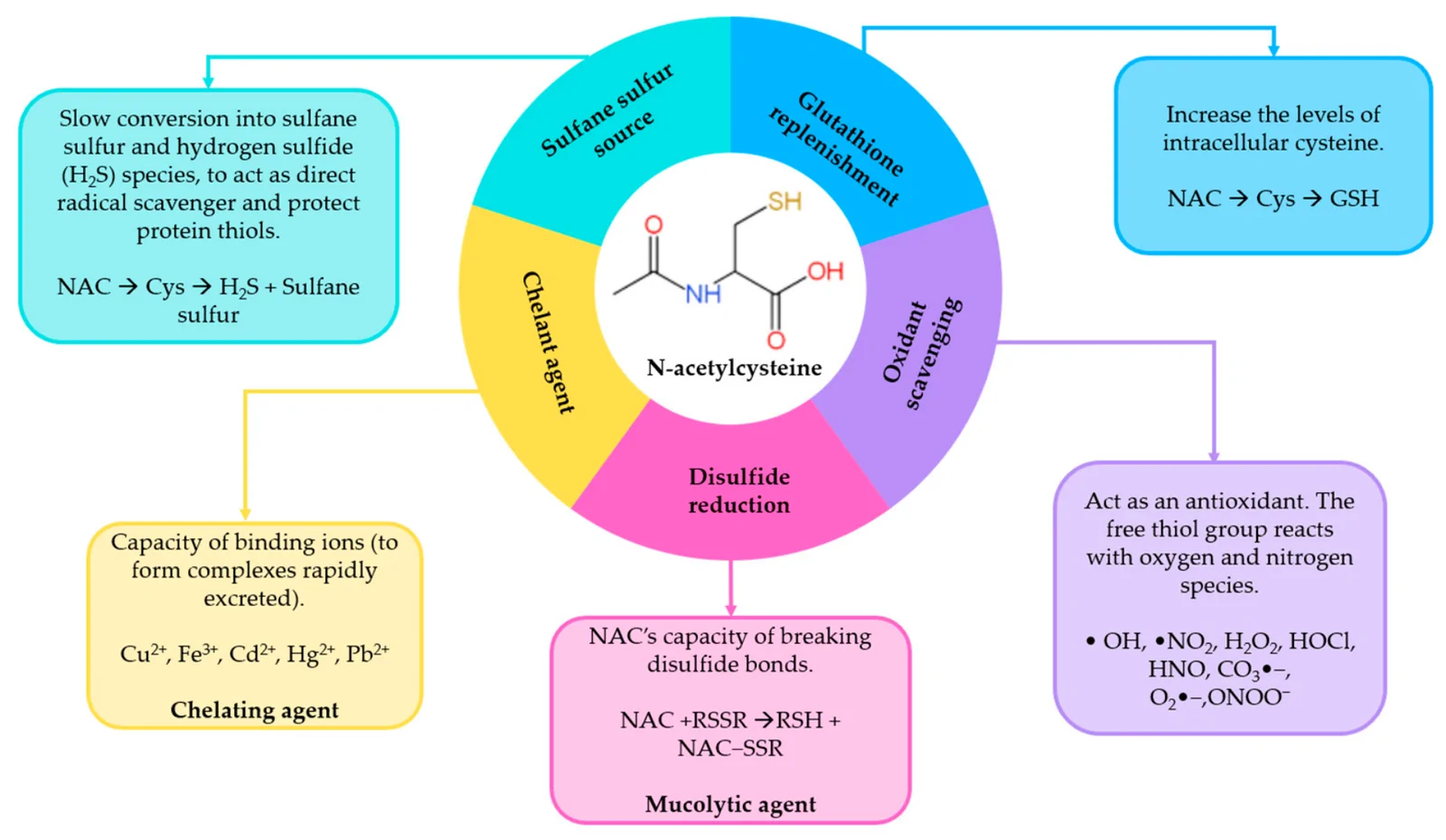
Mechanism of therapeutic action of N-acetylcysteine. Inspired by Tenório et al. (2021) [6] and Pedre et al. (2021) [7].

N-acetylcysteine (NAC) has a wide range of applications, from its use as a mucolytic agent to the treatment of paracetamol overdose [6,7]. Its oxidant-scavenging capacity, antibacterial activity, and role in glutathione (GSH) synthesis [8,9,10,11] have been reported. Studies have shown that NAC can reduce the cytotoxicity of methacrylate and epoxy resin [12] and monomers [13,14]. NAC also exhibits characteristics beneficial for wound healing [15,16], and possesses adhesive qualities [17], highlighting its potential application in hydrogels in the biomedical field. These different applications of NAC arise from three main mechanisms—(1) disulfide reduction agent, (2) oxidant scavenging and (3) glutathione replenishment—and a new emerging mechanism is described as a cysteine prodrug capable of generating hydrogen sulfide (H_2_S) and sulfane sulfur species inside cells [6]. Frequently, NAC’s effect is mostly associated with the antioxidant property because it serves as a precursor to GSH, providing cysteine for GSH replenishment. NAC can also display antioxidant activity independently of GSH synthesis by its conversion to sulfane sulfur species and H_2_S that act as direct radical scavengers and protect protein thiols against irreversible oxidation, shown in Figure 1. The conversion in sulfur species is slower in comparison to cysteine, and as a consequence, NAC is less cytotoxic than cysteine [6]. Additionally, NAC is also capable of binding to metal ions like copper (Cu^2+^), cadmium (Cd^2+^), mercury (Hg^2+^), and lead (Pb^2+^), forming complexes that are eliminated by the organism [7]. Although the NAC potency varies by oxidative stress model, it can be surpassed by agents like vitamin E or BHT [18,19]. However, its safety profile and anti-inflammatory effects make it a valuable tool in biomedical applications.

Although NAC is not universally more potent than other antioxidants, its advantage lies in its multifunctionality. It replenishes glutathione, reduces disulfide bonds, modulates inflammation, and can generate hydrogen sulfide and sulfane sulfur species [6,7,18,19]. Its established clinical use, aqueous solubility, safety profile, and chemically accessible thiol and carboxyl groups further facilitate its incorporation into hydrogels. Thus, its value arises from combined pharmacological and formulation versatility rather than antioxidant potency alone.

In this perspective, it is clear the relevance and applicability of NAC to biomaterials as a molecule of interest. However, NAC has a free sulfhydryl group that is extremely reactive and the application on hydrogel can be a strategy to protect it from oxidation and dimerization.

NAC is an established pharmaceutical ingredient described in the European Pharmacopoeia and Martindale [20,21]. Its hydrophilicity facilitates aqueous formulation but may promote rapid diffusion and limited membrane permeation. Moreover, the reactive sulfhydryl group is susceptible to oxidation and disulfide formation during preparation and storage, potentially reducing active NAC and interfering with quantification [6,7,22]. Hydrogels should therefore provide both controlled delivery and experimentally demonstrated protection against chemical degradation.

Given that (1) NAC suffers from poor oral bioavailability due to its instability in acidic environments and low permeability across biological barriers [23], (2) NAC suffers rapid degradation in conventional delivery methods due to its intrinsic reactivity [22], and (3) high doses of NAC can lead to acute toxicity [24], controlled release systems to target tissues are essential for NAC biomedical applications. Moreover, strategies that protect NAC from premature degradation are necessary to preserve its therapeutic efficacy. In this context, incorporating NAC into hydrogel matrices serves as an effective strategy to enhance its therapeutic potential while enabling sustained release, thereby maintaining optimal therapeutic levels over extended periods.

This systematic review aims to investigate the application of NAC in hydrogels, with particular emphasis on their bioactivity and biocompatibility, as well as the role of NAC as a crosslinker or therapeutic agent in delivery systems. This systematic review was conducted according to the Preferred Reporting Items for Systematic Reviews and Meta-Analysis (PRISMA) guidelines. The SCOPUS, SciFinder, PubMed, and Web of Science databases were searched for this review. The search terms used were: (N-acetyl-L-cysteine OR N-acetylcysteine OR acetylcysteine OR acetyl-L-cysteine) AND (hydrogel* OR biomaterial* OR Sponge OR scaffold OR “material coating”) AND (“in vivo” OR “in vitro” OR “ex vivo” OR cytotoxicity OR biocompatibility OR biocompatible OR “cell viability” OR “cell proliferation” OR “antioxidant activity”). As exclusion parameters, only full articles published, written in English, and published between 2000 and 2025 were included in the review. These parameters were used by automation tools to exclude 188 records from the study.

**Figure 2:**
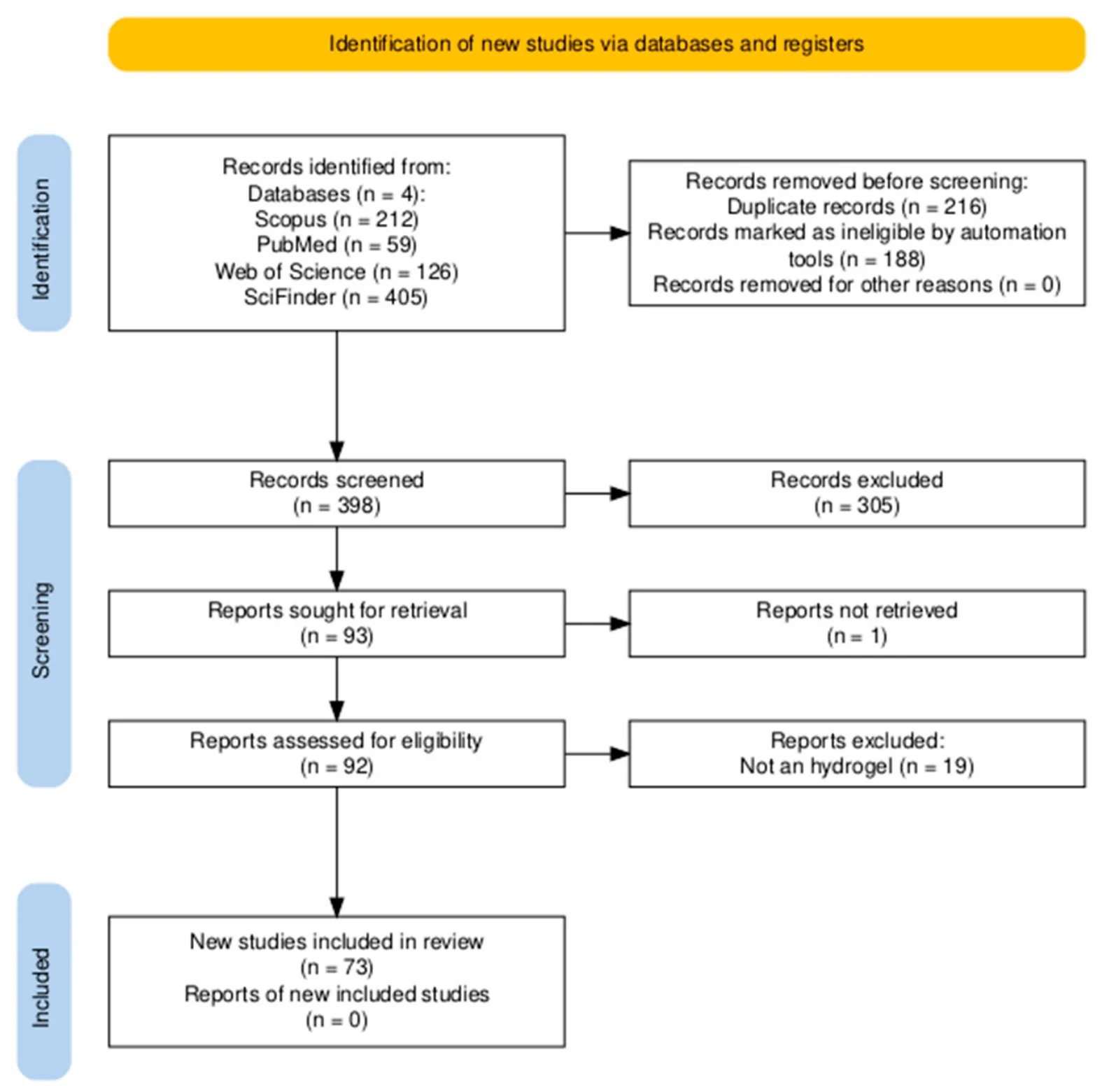
PRISMA diagram created by the authors using the online tool PRISMA Flow Diagram (2026).

In the SciFinder search, the NAC molecule was added and refined with the term “hydrogel”. The PRISMA diagram (Figure 2) displays the process used in this review. Using Zotero as a reference manager, 216 articles were identified as duplicates and removed manually, resulting in 398 screened articles. Based on these criteria, 305 articles were excluded after abstract screening. Of the remaining 93 papers, one could not be retrieved and was subsequently excluded, resulting in 92 articles screened for eligibility. The screening was conducted by one reviewer, who carried it out by reading the abstracts. The amount of literature was randomly divided for reading between two reviewers. In the following step, the articles were read fully by one of the reviewers, independently. During this evaluation, in cases of uncertainty, the decision to include or exclude was evaluated by both reviewers. A third reviewer was consulted to see if the uncertainty remained. A total of 19 studies failed to meet the eligibility criteria because they did not report hydrogel systems. Consequently, this systematic review includes 73 publications that focus on the development and/or characterization of hydrogels that incorporate NAC. This review did not perform a meta-analysis of the data collected, and it did not assess the risk of bias for the studies included (checklist in Supplementary Materials).

## 2. Application of NAC in Hydrogel

**Figure 3:**
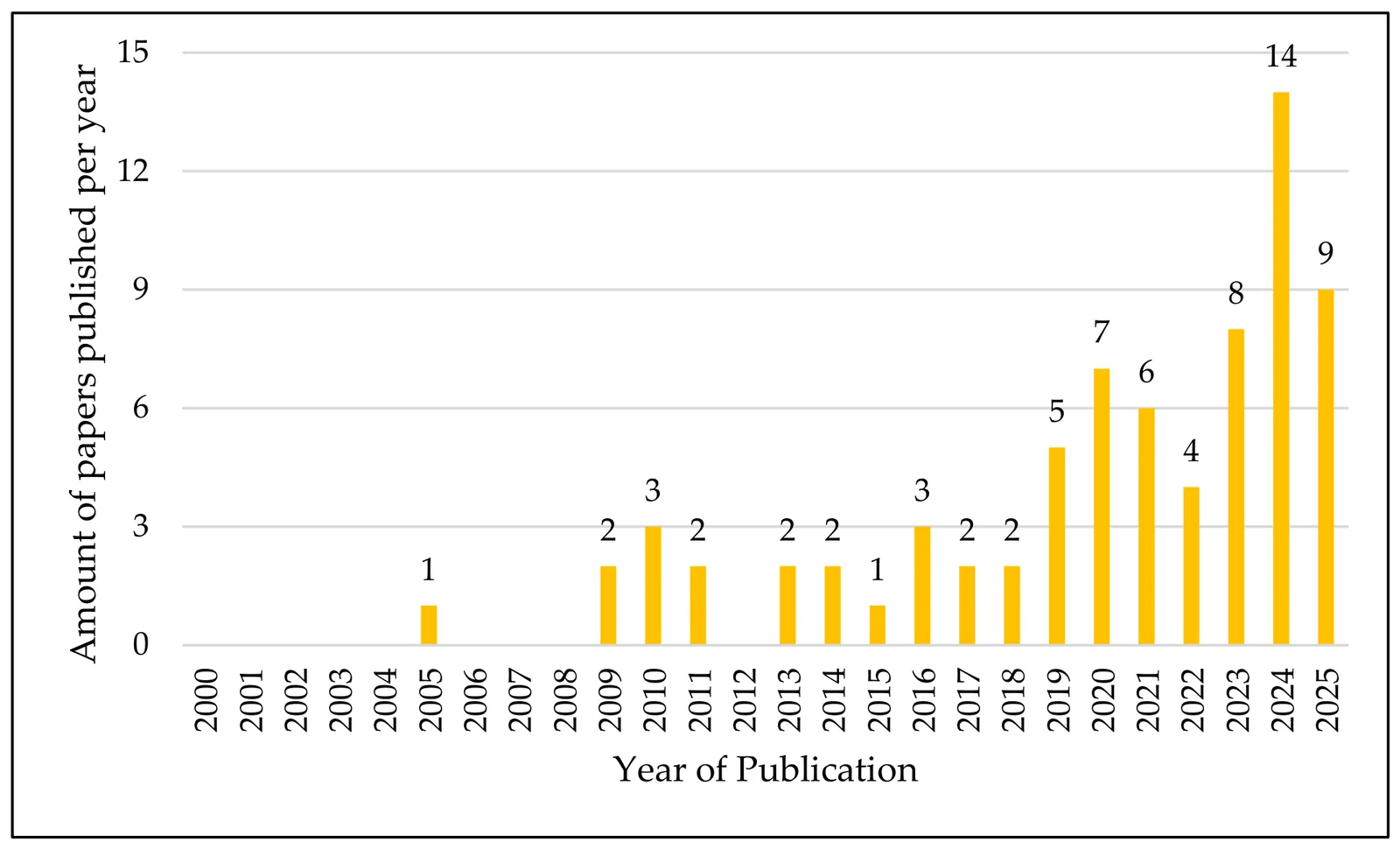
Temporal distribution of published papers on NAC applied to hydrogel systems from 2020 to 2025, with data extracted from SCOPUS, PubMed, Web of Science, and SciFinder.

Between 2000 and 2025, the number of papers published on this subject has increased significantly (Figure 3), demonstrating the growing interest in the potential of NAC applications in hydrogel systems. Most of the articles included in this review employed NAC through different strategic approaches: (i) as a modifying agent conjugated to the polymeric matrix to achieve specific properties; (ii) as a crosslinking agent to improve the physicochemical properties of hydrogels; (iii) as a chelating agent for metal cations; and (iv) as a therapeutic agent loaded directly into hydrogels or incorporated into/onto microparticles dispersed within hydrogel-based controlled-release systems.

**Figure 4:**
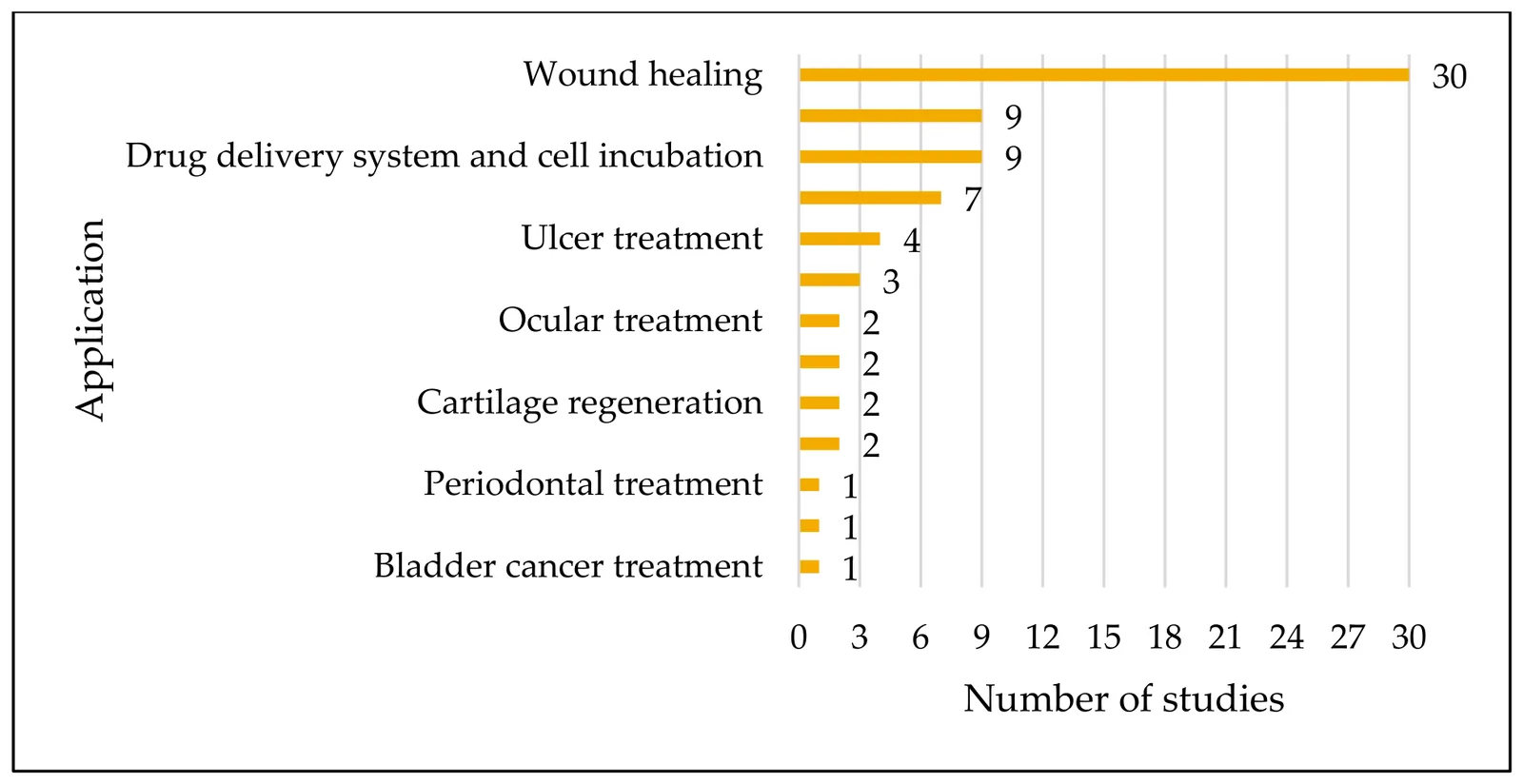
Distribution of published papers of NAC applied to hydrogel systems from 2000 to 2025 regarding its biomedical application.

NAC’s ability to reduce the amount of reactive oxygen species (ROS) has led to its association with accelerated wound healing, collagen deposition, and tissue regeneration, particularly in diabetic animal models [25,26,27]. Hydrogels modified with NAC also demonstrate improved blood clotting and collagen deposition [15]. As is shown in Figure 4, NAC-containing hydrogel systems have diverse biomedical applications. Among the intended applications of NAC in these systems are the development of a wound dressing [10,26,27,28,29,30,31,32,33,34,35,36,37,38,39,40,41,42,43,44,45,46,47,48,49,50], controlled drug delivery system [51,52,53,54,55,56,57,58], liver treatments [59,60,61], ulcer treatment [16,62,63,64] for ocular treatment [65,66], osteoregeneration [49,67,68,69,70,71], brain injury and nerve recovery [72,73], bladder cancer treatment [74], cartilage regeneration [75,76], hearing loss prevention [77,78], periodontal disease [79] treatment, and tissue engineering overall [80,81,82,83,84,85]. Therefore, when integrated into different hydrogel systems, NAC enhances therapeutic potential, demonstrating its versatility and efficacy in various medical applications, as detailed in Table A1 (Appendix A).

## 3. Strategic Approaches to Employing NAC in Hydrogels

**Figure 5:**
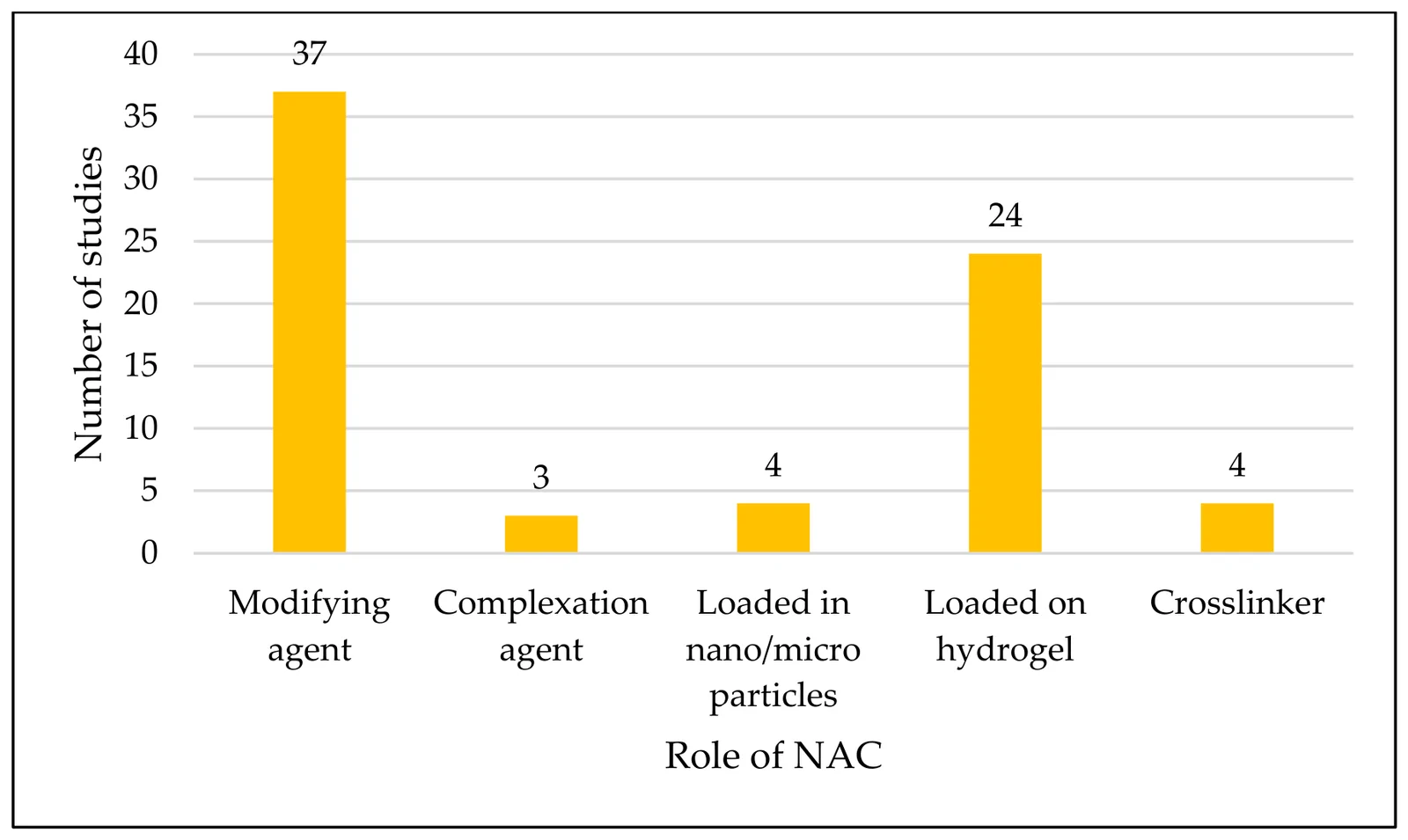
Distribution of published papers using different strategic approaches to employing NAC in hydrogel systems from 2000 to 2025.

The strategies for using NAC on hydrogels reported in the literature range from the simple loading of NAC [10,70] into the hydrogel solution before gelation, to develop delivery systems, to covalent functionalization of the polymeric chains with NAC [31,45,46,51,64], encapsulation as a therapeutic substance in nano- and microparticles and dispersion in a hydrogel [27], and use as a crosslinking agent [53,65,82,84]. Figure 5 shows the number of papers that used these different strategic approaches.

To categorize the application strategies of NAC within the evaluated hydrogel systems, it is essential to define the distinct roles identified in the literature compiled in this review. Several studies have used NAC as a modifying agent to functionalize polymer chains via covalent bonds, thereby adding new functional groups to the matrix. NAC is frequently added as a pendant group through a chemical reaction using the 1-ethyl-3-(3-dimethylaminopropyl)-carbodiimide hydrochloride/N-hydroxysuccinimide (EDC/NHS) system under acidic conditions. The addition of NAC to the polymeric chain can also promote its second role as a crosslinking agent due to hydrogen and disulfide bond formation between the chains or even S-S bonds formed by the air oxidation of free thiols. Owing to the functional groups of the NAC molecule, it is possible to form covalent bonds through the carboxyl groups with the amide groups of polymers such as chitosan [30,31,45,46], carboxymethyl chitosan [25,64], glycol chitosan [74], hyaluronic acid [51], and gelatin [80], etc. A pair of publications used NAC to prepare a new crosslinker. In these cases, the NAC acts as a chemical reagent. A few studies have produced inorganic hydrogels, where the gel network is a product of the chemical bonds formed between NAC and a metal ion, generating a coordination complex. NAC’s role is, therefore, that of a complexation agent [35,86]. Multiple studies have used NAC loaded into hydrogel networks to develop drug delivery systems. In a small number of studies, NAC was loaded onto particles and incorporated into hydrogels.

Each approach can be tailored by controlling the hydrogel formulation, which depends on the intended application of the material. When the goal is to develop drug delivery systems, most studies apply NAC loaded into hydrogels or onto particles for drug delivery, as summarized in Table A1 (Appendix A). Conversely, NAC is also used as a polymer-modifying agent to enhance its physical-chemistry properties. These include reducing the hydrophobicity of the material, enabling self-healing, and improving its adhesiveness, biocompatibility, and bioactivity.

### 3.1. N-Acetylcysteine-Modifying Agent for Hydrogel Applications

**Figure 6:**
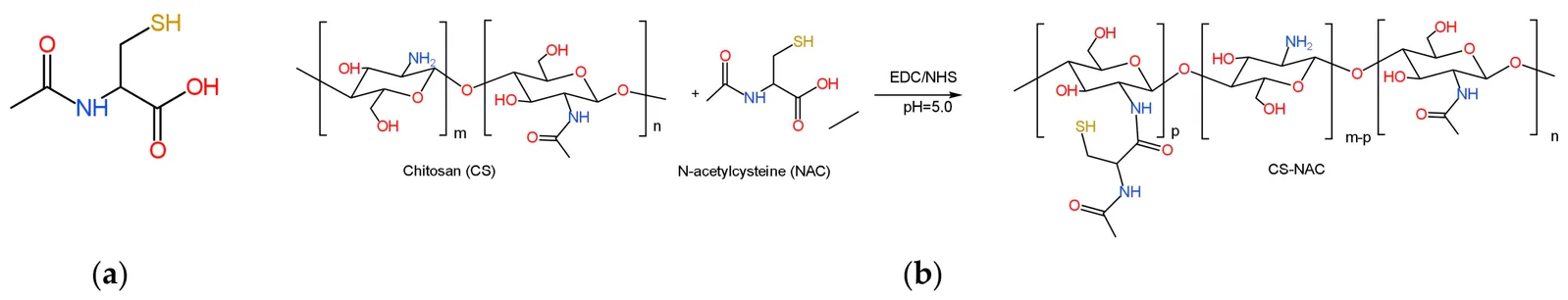
(**a**) NAC molecular structure, (**b**) Chitosan functionalization with NAC mediated by carbodiimide. This figure was made using KingDraw Chemistry Station v3.6.1.

NAC possesses two nucleophilic and reactive functional groups capable of undergoing chemical reactions with polymer chains: the thiol (sulfhydryl, -SH) group and the carboxylic acid (-COOH) group (Figure 6a). NAC can be conjugated to the polymer chains that compose the hydrogel matrix, such as chitosan, via an amidation reaction, as shown in Figure 6b. The amine from the polymer reacts and forms a covalent bond with the carboxylate group of the NAC molecule [31].

Several studies have functionalized polymers with NAC for various reasons, such as decreasing hydrophobicity, enhancing adhesiveness, trapping ions such as silver, increasing crosslinking density, and adding specific trigger responses. Yang et at. [46] introduced NAC-modified chitosan (CS-NAC) as an anchor to nitric oxide and a source of imine bonds in the hydrogel. The gelation time decreased with increasing CS-SNO concentration, demonstrating the crosslinking effect of the imine bonds provided by NAC modification via the Schiff reaction. Because of the dynamic imine bonds, gelation was pH-responsive, and the hydrogel was formed under neutral and basic conditions. Additionally, nitration of sulfhydryl groups produces a nitric oxide donor, thereby improving the antibacterial activity of the material [46].

Zheng et al. [87] developed a hydrogel to treat diabetic wounds, in which NAC was chemically conjugated to the polymeric matrix, composed of cysteine-modified sodium hyaluronate (HA-SH), a copolymer (PDA-PEG) formed by the polymerization of pyridyl disulfide acrylate (PDA) and poly(ethylene glycol) methyl ether methacrylate (PEGMA). To PDA-PEG, NAC and bFGFs were conjugated by -S-S bond, via oxidation. The outcome was an intelligent ROS-responsive hydrogel. Therefore, NAC and bFGF release was higher in oxidative or acidic media than in PBS or water [87].

### 3.2. N-Acetylcysteine as Crosslinker in Hydrogel Systems

**Figure 7:**
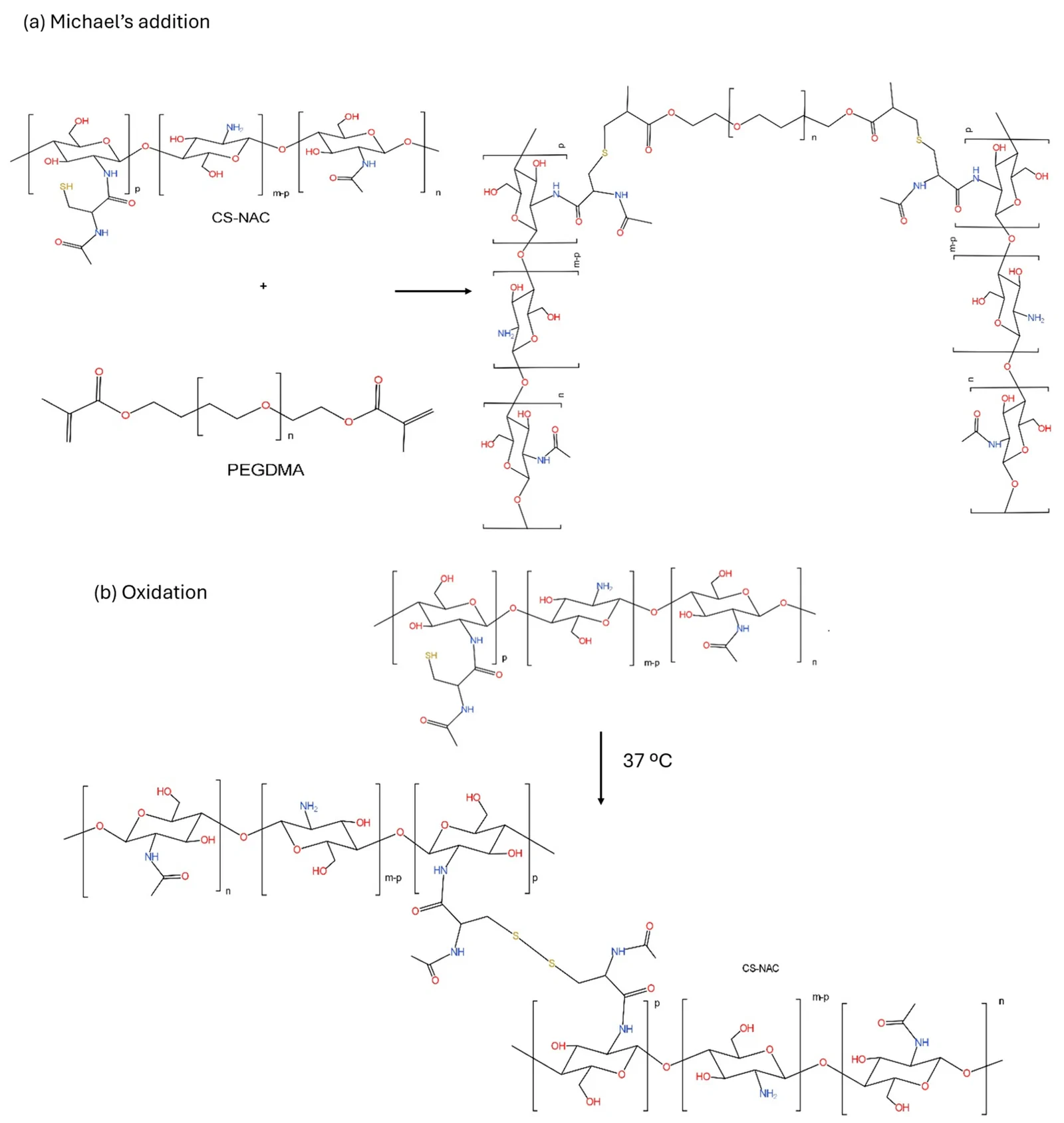
(**a**) Representation of crosslinking between CS-NAC and PEGMA via Michael’s addition, (**b**) CS-NAC crosslinking via thiol oxidation. This figure was made using KingDraw Chemistry Station v3.6.1.

As previously stated, NAC can act as a crosslinker, even when conjugated into a polymeric chain, because of the free thiol. Gelation was triggered by a rapid thiol–ene reaction between the thiol group and the double bond (C=C), forming a C-S bond and, in the process, a three-dimensional network was obtained within seconds (Figure 7a). When the double carbon bond is activated, the reaction is called Michael addition (Figure 7) [40,44,48,53,56,75,81,83].

NAC can form crosslinks via the oxidation of thiol groups, leading to the formation of S-S bonds between polymeric chains (Figure 7b). Free thiol groups also play a role in gelation via disulfide bridges [37,51,57]. Bermejo-Velasco et al. [51] prepared a pH-responsive hydrogel from sodium hyaluronic acid modified with NAC, in which gelation occurred in neutral and basic pH environments and was faster at higher pH values. At a basic pH, the acidic hydrogen from the sulfhydryl group of NAC dissociates, facilitating the formation of disulfide bonds. For comparison, the gelation of the polymeric matrix modified with cysteine occurred within 3.5 min (pH 7.4 and 11% degree of substitution (DS)), and the modification with NAC occurred within 10 h (DS of 9%, pH 7.4). The difference in gelation time at pH 7.4 can be attributed to steric factors, as highlighted by the authors. Because gelation depends on S-S bond formation, it is also dependent on the pKa of the –SH group, which is 7.0 for cysteine and 9.35 for NAC [51].

Nie et al. [40] obtained a hydrogel by mixing a CS-NAC solution and polyethylene glycol-maleimide modified polypeptide crosslinker (EPL-PEG-MAL) at a physiological pH within less than 1 min. The gelation time achieved by the thiol–ene click reaction was shorter than that achieved by the spontaneous oxidation of thiol groups. The authors reported that the gelation time decreased from 35 s to 7 s with an increase in the amount of PEG-maleimide available to react with CS-NAC. The density of crosslinking reflected in the mechanical properties; the storage modulus increased from 300 Pa to 1614 Pa [40]. Teng et al. [81] tested a self-assembly hydrogel by crosslinking CS-NAC and PEGDA at 25 °C and 37 °C in PBS. The authors concluded that the gelation time was shortened under physiological conditions, which is desirable for such applications. An increase in CS-NAC concentration decreased the gelation time [81]. Michael’s addition as a crosslink approach is possible in mild conditions (low temperature, pH 7.2–7.4); it is fast and does not produce oxidative species that can cause damage to biological tissues, and by so doing, impact the cytocompatibility of the materials prepared.

### 3.3. Hydrogel Formation via N-Acetylcysteine–Metal Complexation

In some cases, NAC was used not only as a crosslinker but also as a major component, forming a hydrogel from nanoclusters via a complexation reaction with silver ions [86,88].

NAC and cysteine can form a coordination complex with Ag ^+^ ions and reduce them. Gelation occurs through the interaction of the partly protonated carboxyl groups of NAC/Ag^+^ via hydrogen bonds, encapsulating the reduced silver ions [86]. Vishnevetskii et al. [86] showed the presence of a wide band at 280–310 nm UV spectra, related to the ligand-to-metal charge-transfer transition. The disappearance of S-H bands on the infrared spectrum after the silver addition and the appearance of the S-S band at 550–500 cm^−1^ are taken as evidence of the coordination reaction [86]. The NAC/Ag^+^ hydrogel is of interest in the development of materials with antibacterial properties, as hydrogel stability is sensitive to physiological pH, which leads to the release of both components, known for their activity against microorganisms.

Laxmanan et al. [88] prepared an inorganic hydrogel by coordinating NAC to silver nitrate. The authors added polyethylene glycol diacrylate (PEGDA) to enhance structural stability and tannic acid (TA) due to its potential to enhance fibroblast proliferation [88]. The peak at 536 cm^−1^ in the FTIR spectra indicates the formation of an Ag-S bond. The formulations containing PEGDA showed a peak at 1111 cm^−1^ attributed to the S-C bond, indicating the successful crosslinking of NAC with PEGDA. TA also interacts with PEGDA and NAC via hydrogen bonds. The formulation of only the inorganic matrix (NA) displayed high antibiofilm capacity against *S. aureus* and good antioxidant efficacy, which the authors attributed to its lower degree of crosslinking, which facilitated the release of silver salts. It also showed an inhibition zone against *S. aureus* and *E. coli.* These findings showcase a different and synergistic approach to designing new materials.

## 4. Sustained Release of NAC from Hydrogels for Therapeutic Applications

It is important to produce new materials capable of promoting controlled drug release. In this scenario, hydrogels play a role because of their capacity to absorb a substantial amount of water and sustain controlled release in situ [3]. The release of NAC from hydrogels can be controlled by diffusion through the polymeric matrix, matrix degradation, desorption equilibrium when the drug is immobilized on particle surfaces, and a responsive trigger when it is tailored into the polymeric chains [10]. The therapeutic interest in developing controlled-release systems for NAC arises from its effectiveness in microbial control, tissue repair, angiogenesis, and cell protection.

Conventional in vitro assays quantify NAC transfer from the hydrogel into an artificial medium, whereas Franz cell studies evaluate transport across a membrane or biological tissue. Ex vivo retention assays measure NAC remaining within tissue compartments, while in vivo studies additionally incorporate clearance, metabolism, tissue binding, and vascular absorption [26,27,29,39]. These outcomes should not be compared interchangeably, and experimental reports should specify the membrane, receptor medium, sampling protocol, assay duration, and maintenance of sink conditions.

**Figure 8:**
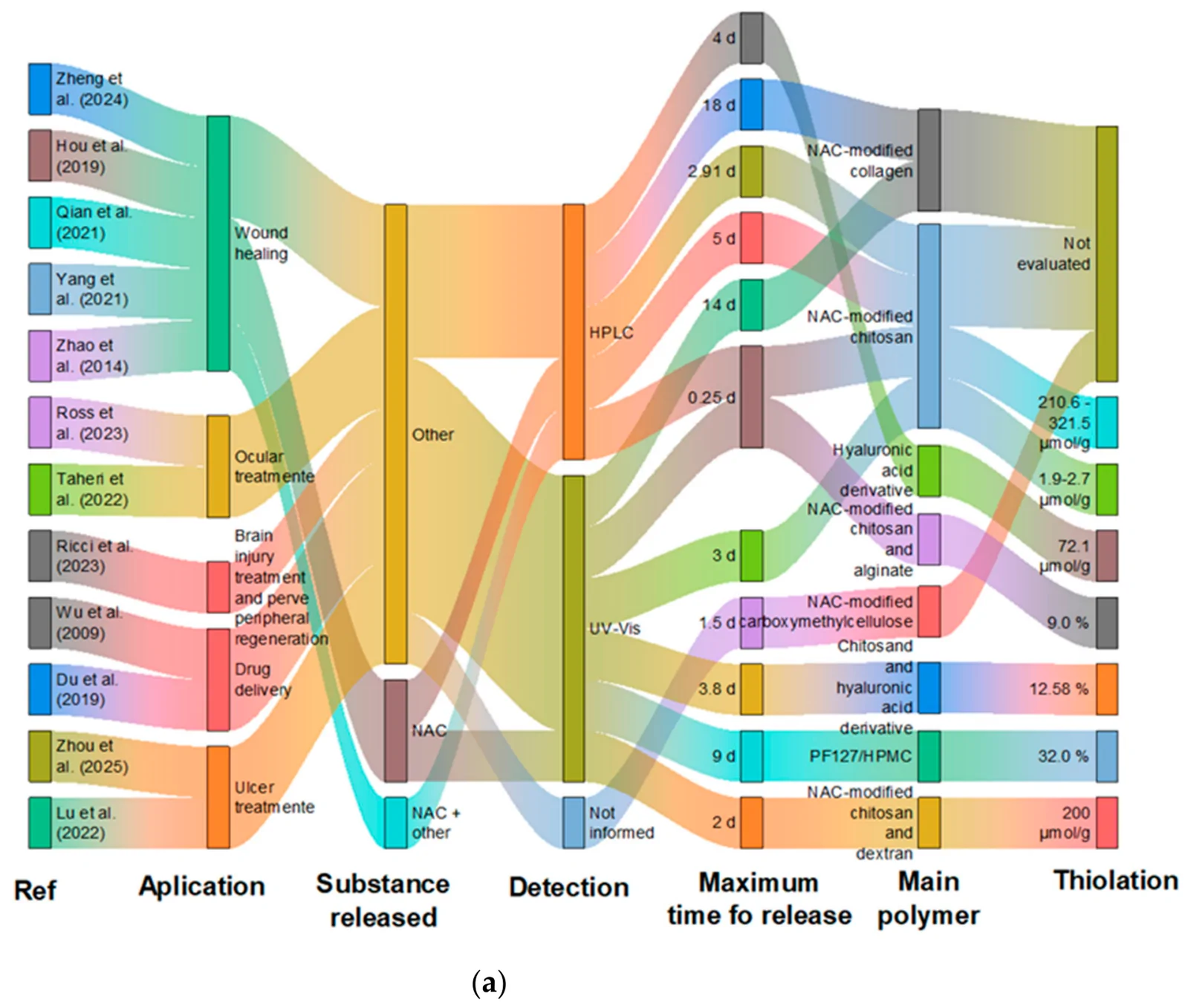

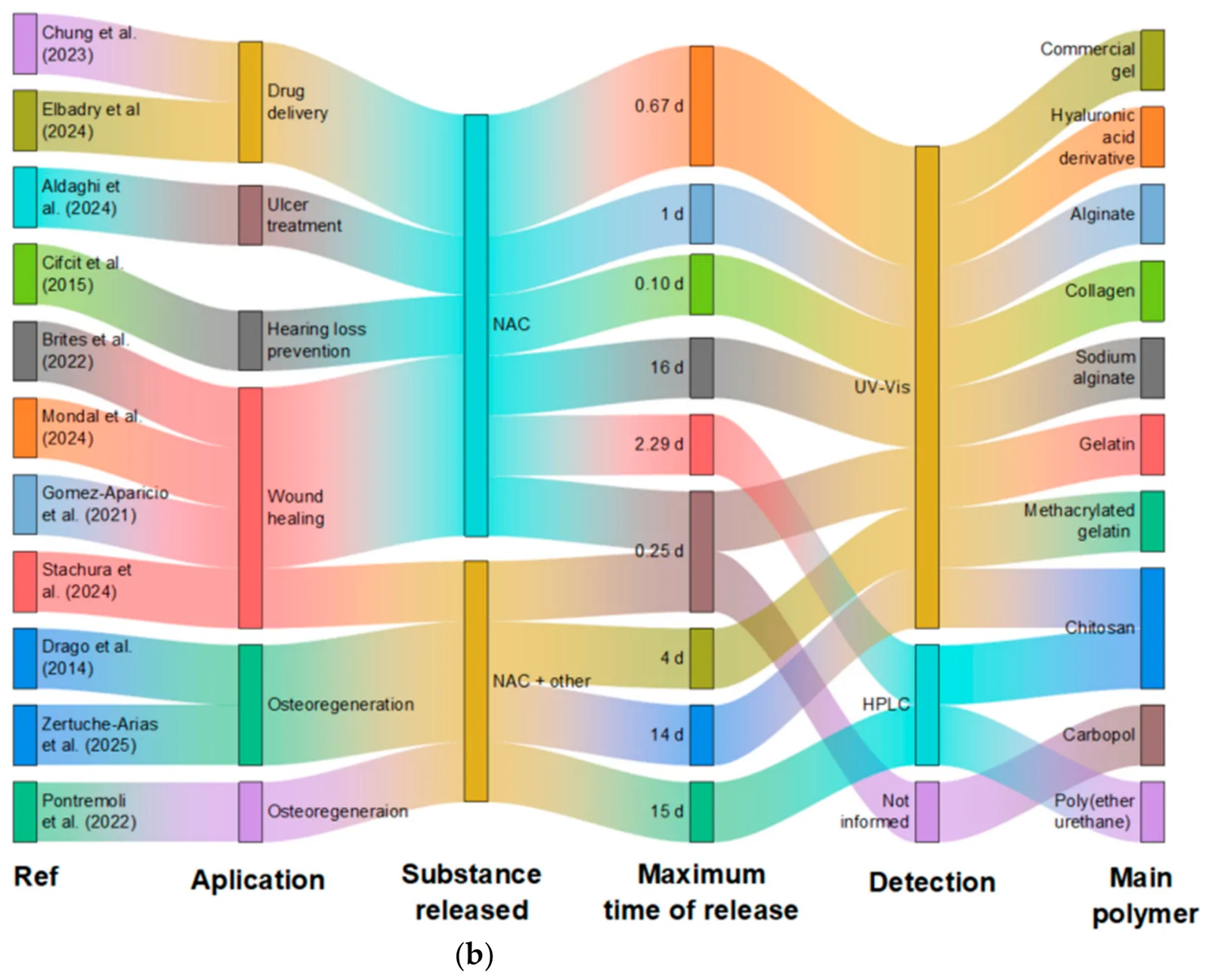
Sankey diagram illustrating the relationships among selected articles regarding target application of the NAC-loaded hydrogel, monitored released substances, analytical instruments utilized, maximum release duration, main polymer matrix when NAC was loaded in the hydrogel (**a**) and, thiolation degree when NAC was used as polymer modifying agent (**b**). The references cited are [15,26,27,29,33,39,46,49,50,52,54,58,62,63,64,65,66,68,70,74,77,87,89].

From the 73 studies evaluated, 23 performed the release of a drug (mostly NAC), ions, peptides, or growth factors, and Table 1 summarizes the main findings. To better visualize the dataset, two Sankey diagrams (Figure 8a,b) correlate the intended hydrogel application, the released therapeutic substance, maximum release duration, analytical detection equipment, main polymer base, and, when applicable, the degree of thiolation achieved via NAC polymer modification. Within this universe of studies, 14 studies reported NAC release, of which 11 targeted NAC for release (loaded into the hydrogel). This represents 19% of the total studies selected in this review. Based on these findings, it is notable that NAC is primarily intended not as a delivered drug, but as a key component for hydrogel modification to enhance its physicochemical properties. The techniques used to quantify NAC are HPLC [29,33,36,87] and UV-Vis spectrophotometry [15,52,57,59,89], being relatively more accessible than HPLC. Although most literature reports calibration curves and working ranges, comprehensive analytical validation parameters remain frequently unreported.

As can be seen in Table 1, the degree of swelling varies greatly from one study to another, as does the composition of the hydrogels. Even within a single article, there is considerable variation in these experimental data on the degree of swelling, depending on the formulation of the hydrogel preparation [26,39,50]. This heterogeneity makes it difficult to identify the conditions that may affect the release of the loaded drugs. Another discrepancy lies in the duration of the release assays, which range from 6 h to 18 days. The duration of the assay depends of the release capacity and hydrogel stability, directly influenced by its chemical composition.

Among the studies that evaluated the release mechanism, it was noted that the transport behavior of the drugs from the hydrogel into the surrounding medium is described as non-Fickian diffusion [20,22,32,42]. Thus, release occurs through hydrogel swelling, followed by erosion and diffusion. Brites et al. [29] observed the same behavior for both NAC and lysine release, showing that in this case, the release behavior was governed by the polymeric matrix. Zhao et al. [50] also demonstrated that the interaction between the drug and the polymeric matrix is fundamental to the release behavior. As the content of maleic acid-modified dextran increased, the loaded drug was released more slowly, especially during the first few hours, when hydrogel degradation is not significant enough to influence the release process [50].

Stachura et al. [27] demonstrated that the release medium also affects the release rate; NAC is released more rapidly in PBS at pH 5.5 than at pH 7.4, while maintaining the same non-Fickian transport behavior. The release of NAC and the concentration of NAC carried—ranging from 5 to 20% wt—did not affect the release behavior; lower concentrations demonstrated better performance in wound healing in the selected animal model.

It can be observed that the amount of released NAC was quantified even when it was covalently bound to the polymeric structure of the hydrogels (Figure 8a), confirming that its release does not depend solely on the diffusion process, but also on the degradation of the polymeric networks. However, it is difficult to make a quantitative comparison, since the thiolation degree was not quantified in all studies; among those that did quantify it, the data are presented in different units (percent or μmol/g).

Among these studies, nine evaluated wound-healing applications, including an ex vivo skin permeation assay performed with human surgical skin in Franz diffusion cells [29]. Although the release assays indicated that drug transport is governed by concentration gradients, diffusion, and swelling degree, no evidence of NAC skin permeation was observed despite its successful release from the Carbopol hydrogel, an outcome attributed by the authors to insufficient analytical sensitivity.

Alternatively, in another study rat skin was employed ex vivo [90] to assess the bioadhesive properties of the hydrogel formulations. Additionally, Mondal et al. (2024) [39] evaluated NAC release from hydrogels synthesized by combining alginate and guar gum. They also investigated NAC permeation through an ex vivo goat skin model using Franz diffusion cell. The authors observed that the drug encapsulation efficiency reached 97% with an increased crosslinker concentration. The optimal formulation exhibited a non-Fickian release mechanism, achieving 100% ex vivo drug permeation within 24 h.

Overall, through these studies, it is possible to highlight the intrinsic analytical challenges of release and permeation assays, particularly regarding the need to establish method detection limits, address NAC oxidation or degradation during testing, and account for incomplete recovery or non-specific adsorption onto hydrogel polymers, membranes, and experimental apparatus.

In drug delivery systems, the swelling behavior of the hydrogel, which is regulated by the network formed by a polymeric matrix, can regulate the delivery kinetics of the active substance [29,91]. High crosslinking bonds degree typically reflect lower swelling and water sorption. This property can be modulated according to the matrix composition. Yi et al. [10] produced a pH/ROS-responsive hydrogel, composed of aldehyde-functionalized hyaluronic acid, to act as a drug delivery system for wound and tumoral treatments containing NAC, 5-fluorouracil (5-FU) and 5-aminolevulinic acid (ALA). Swelling, pore diameter, and gelation time were assessed at different molar ratios of modified hyaluronic acid to thioketal-based linker. Because gelation is triggered chemically by the formation of dynamic imine bonds between the aldehyde and amine groups, an increase in the amine content leads to rapid gelation, less homogeneous pore sizes, and stiffer hydrogels [10].

Li et al. [36] developed a hybrid membrane by incorporating graphene oxide (GO) into a collagen matrix and loading it with 0.1 mg/mL NAC mixed with the carbodiimide crosslinker agent, EDC/NHS. NAC was bonded by amide in the collagen structure, while the thiol group was free, as indicated by the presence of -SH stretching band at 2549 cm^−1^ in the Raman spectrum. The authors noted that the addition of NAC did not affect the pore size, elastic modulus, or structure of the hydrogels, as shown by the SEM images. In contrast, the release of NAC was rapid in the first 4 h, which was attributed to the free NAC deposited in the pores of the membranes, followed by a sustained and slower release triggered by the degradation of the membrane network, where the chemical bonds were broken [36]. The release of NAC was much slower in the groups containing GO because the pore structures were better distributed and interconnected.

**Figure 9:**
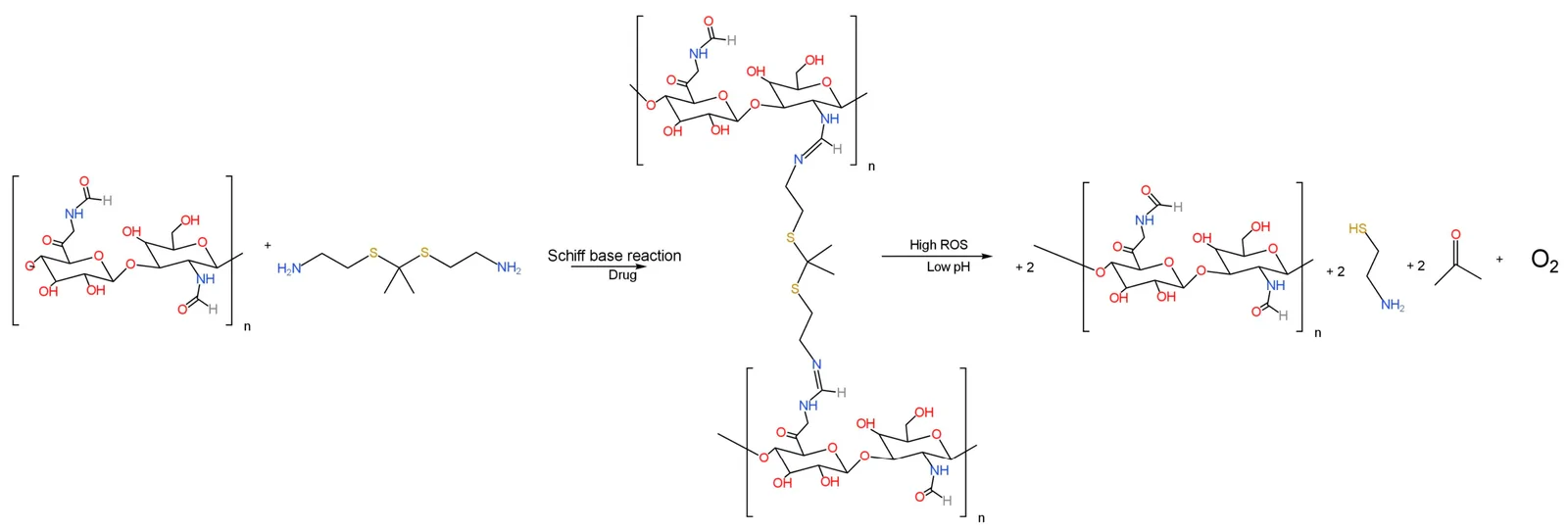
Schiff base reaction. This figure was made using KingDraw Chemistry Station v3.6.1 inspired by ref [10].

Yi et al. [10] developed a dressing hydrogel for burn wound therapy that released NAC via the Schiff base reaction. The hydrogel is composed of aldehyde-modified hyaluronic acid (SA-HA) and a thioketal linker with amino end groups. Cross-linking bonds form between the amino end groups and aldehyde, as shown in Figure 9, which are broken in acidic conditions and high concentrations of reactive oxidative species (ROS) medium, releasing the encapsulated NAC. The authors discovered that 86.86% of the NAC encapsulated was released in 6 h 30 min in a pH 5.5 and H_2_O_2_ environment, which is four hours faster than in physiological pH, confirming its responsive nature. A burst release occurs within the first three hours, followed by gradual release patterns [10].

Qian et al. [26] loaded and crafted NAC via amidation into a chitosan and graphene oxide hydrogel system, and demonstrated a sustained release for 18 days. The burst release in the first 24 h occurred due to free NAC, followed by a slow increase in NAC release, most likely due to degradation and breakage of the covalent bonds formed between the scaffold and NAC [26]. In another study, NAC encapsulated in a GelMA/ChMA hydrogel demonstrated a burst release of approximately 50% of the cumulative quantity of NAC in PBS at pH 7.4 within the first 48 h, followed by a sustained release period of approximately 90% in 7 days. After 14 days, the hydrogel was completely degraded, which was accelerated by the presence of proangiogenic peptides. The release was most likely controlled by the swelling, diffusion, and degradation of the polymeric chains [49].

Pontremoli et al. [70] prepared a thermosensitive hydrogel for the co-release of Sr^2+^ ions and NAC by adsorbing NAC onto mesoporous bioactive glass particles. The hydrogel obtained showed a sol–gel transition at 25 °C, and the injectability was not affected by the presence of nanoparticles; the thermosensitivity was a result of gelation only. The incorporation of these particles into the hydrogel displayed sustained release, reaching 90% cumulative release over 7 days in Trizma [70].

Cross-study comparison indicates that NAC incorporation strategy is more influential than polymer identity. Physical loading commonly produces an initial burst, whereas covalent or stimulus-responsive association generally enables more controllable release [26,27,33,70,77,87]. Wound healing currently has the strongest preclinical evidence [15,16,26,27,33,36,39,62], while other applications rely on fewer heterogeneous studies. No polymer class repeatedly failed; recurrent limitations were burst release, incomplete stability assessment, short observation periods, and insufficient dose–response evaluation.

These studies are some examples that highlight how hydrogel systems can act as a delivery system to NAC. Overall, delivery systems where NAC is chemically bonded to the polymeric matrix provided longer sustained release than when it is only loaded into hydrogel or onto micro/nanoparticles. In summary, some studies have investigated the controlled release of NAC achieved by loading it within a hydrogel, with a focus on a trigger-responsive approach, especially because of the chemistry of the injury environment, which is rich in oxidative species as a response to inflammation mechanisms.

Some of the studies considered in this review have quantified NAC release using ultraviolet-visible (UV-Vis) spectrophotometry, due to its characteristic absorption in the UV region. However, analytical protocols must account for potential degradation products formed during both the hydrogel preparation and analysis stages. Commercial NAC may contain three major impurities: L-cystine, L-cysteine, and N,S-diacetyl-L-cysteine, for which the European Pharmacopoeia stipulates a maximum limit of 0.5% [20,22]. Additionally, N,N-diacetylcystine can be formed during storage, particularly under stress conditions such as elevated temperatures (e.g., 40 °C) combined with high relative humidity (~75%). Subsequent degradation can release hydrogen sulfide, imparting a characteristic sulfurous odor. Consequently, the potential degradation of NAC must be carefully evaluated during hydrogel preparation, necessitating the use of validated analytical techniques [92], such as high-performance liquid chromatography (HPLC).

## 5. Biocompatibility

The application of hydrogels in the biomedical field, such as in drug delivery and tissue regeneration, requires these materials to exhibit high biocompatibility, ensuring adequate interaction with the biological environment, minimizing unwanted immune responses, and ensuring their safe elimination without compromising the integrity of the organism. In this context, the incorporation of NAC has been widely investigated because of its ability to modulate cellular and inflammatory processes, favoring the biological compatibility of biomaterials [34,38,73]. Several studies have demonstrated that materials containing NAC exhibit good interactions with different cell types, preserving cell viability and, in some cases, promoting biological events associated with tissue regeneration [34,38,73].

The analysis of the studies compiled in Table A1 demonstrates that cell viability assays are the most frequently employed approach to evaluate the biocompatibility of biomaterials containing NAC. These evaluations encompassed different cell lines, including fibroblasts, keratinocytes, endothelial cells, mesenchymal stem cells, osteoblasts, and macrophages [17,29,57,79,81,84,86,90]. Overall, the results showed high biological compatibility, as reflected by the maintenance of cell viability in different experimental models. Live/Dead assays corroborated these findings by demonstrating a predominance of viable cells after exposure to biomaterials [10,25,30,32,34,35,36,48,49,50,53,55,56,57,75,80,93,94].

Although less frequently investigated, hemocompatibility is an important parameter for evaluating the biosafety of biomaterials, especially in applications involving contact with body fluids and vascularized tissues [10,25,30]. Hemolysis tests reported in the literature indicated compatibility with blood components, suggesting that the incorporation of NAC does not compromise the integrity of red blood cells [39]. In this context, Moldal et al. (2024) [39] evaluated a sodium alginate and guar gum-based hydrogel containing NAC for the treatment of diabetic wounds and observed a hemolytic potential of 3.92 ± 0.17%, a value considered within the acceptable limits for biocompatible materials. In addition, assays using human gingival fibroblasts (HGF-1) demonstrated adequate cell adhesion and proliferation on the hydrogels, indicating that the material provides a microenvironment favorable for cell growth [39].

In vivo studies also play a fundamental role in evaluating biocompatibility, as they allow for the simultaneous investigation of biomaterial degradation, local inflammatory response, and interaction with complex tissues. In general, materials containing NAC have shown good biological tolerability, with no significant evidence of adverse reactions. A representative example was reported by Kong et al. (2025) [17], who developed an asymmetric adhesive fiber membrane composed of GelMA-Gel/NAC for hemostatic applications [17]. In biocompatibility and biodegradation tests performed on rats, the biomaterial was completely absorbed after 30 days, accompanying the healing process without compromising tissue integrity [17]. Histological analyses did not reveal significant infiltration of inflammatory cells, whereas the expression of the pro-inflammatory cytokines TNF-α and TNF-β was significantly reduced compared to that in the control group. These results suggest that the incorporation of NAC not only preserves the biocompatibility of the material but also contributes to the modulation of the inflammatory response during tissue repair [17].

## 6. Bioactivity

The bioactivity of hydrogels containing N-acetylcysteine is closely related to the ability of the molecule to act as a precursor to glutathione and a scavenger of reactive oxygen species (ROS) [6,89]. By modulating oxidative stress and inflammatory responses, NAC can influence several biological processes relevant to biomedical applications, including microbial control, tissue repair, angiogenesis, and cell protection [6,15,17,89]. Therefore, the incorporation of this molecule into hydrogels has been explored to improve the biocompatibility of materials and confer therapeutic functionalities capable of enhancing their clinical efficacy.

### 6.1. Antioxidant Activity and ROS Scavenging

Many formulations contain chitosan, graphene oxide, metallic ions, peptides, growth factors, or photothermal agents that may independently contribute to biological activity [10,25,32,35,37,42,61,78,87,88]. Consequently, the performance of a complete formulation cannot automatically be attributed to NAC. Matrix-only, free-NAC, co-agent-only, matrix–NAC, and complete-formulation controls are required to distinguish NAC-specific effects from additive or synergistic responses. Without these controls, outcomes should be attributed to the multifunctional system rather than to NAC alone.

Oxidative stress results from an imbalance between the production of reactive oxygen species (ROS) and the body’s antioxidant capacity and is associated with cell damage, persistent inflammation, and impaired tissue repair processes [6,89]. In this context, N-acetylcysteine (NAC) stands out as a potent antioxidant agent because of the presence of thiol groups (-SH) in its molecular structure, which can react directly with reactive species [89]. In addition, NAC acts as a precursor to glutathione (GSH), one of the main endogenous antioxidants, contributing to the restoration of cellular redox homeostasis and protection against oxidative damage [6,89].

Addressing topical skincare applications, poly(methyl methacrylate) (PMMA) and marine collagen nanofibers loaded with NAC were developed for topical application and evaluated for their antioxidant activity [89]. The optimized formulation (3PMMA:1MC/10% NAC) showed high activity of the antioxidant enzymes superoxide dismutase (SOD) and catalase (CAT), reaching 420 and 744 mU mg^−1^, respectively. This effect was attributed to NAC’s ability to preserve SOD activity by preventing peroxynitrite-induced nitration of tyrosine residues, an important mechanism associated with the inactivation of this antioxidant enzyme. Furthermore, DPPH free radical scavenging assays showed high antioxidant capacity for both free NAC and the formulation containing NAC, confirming the effectiveness of incorporating the molecule in maintaining its free-radical-scavenging activity. These results demonstrate that the incorporation of NAC into hydrogel systems can significantly contribute to protection against oxidative damage, favoring applications aimed at tissue repair and regeneration for facial mask development, for example [89]. In another in vitro study, a carbomer-based hydrogel loaded with NAC and L-lysine acted as a chemical glove by remaining on the skin surface to sequester allergens and providing a non-irritating and non-sensitizing protective layer [29].

Cisplatin is a chemotherapeutic agent widely used in the treatment of various neoplasms; however, its clinical application is frequently limited by the adverse effects associated with oxidative stress, including ototoxicity [77,78]. To mitigate these effects, Ge et al. [78] developed a system based on nanozyme NAC-derived carbonized polymer dots embedded in a Zr-organic frame-Mn (Zr-MOF-Mn) mesoporous structure coated with polydopamine (PD) and loaded in a photopolymerized methacrylated gelatin hydrogel (GelMA). The antioxidant activity of the system was confirmed using different assays, which demonstrated a high capacity for scavenging reactive oxygen species, including superoxide anions (O_2_•^−^) and hydroxyl radicals (•OH). The results showed a synergistic effect between the components of the nanozyme, resulting in superior performance compared to that of the isolated constituents. In HEI-OC1 cells subjected to cisplatin-induced injury, a hydrogel loaded with nanozymes significantly reduced intracellular and mitochondrial ROS levels while preserving the mitochondrial membrane potential, indicating protection against mitochondrial dysfunction induced by oxidative stress [78]. Additionally, Western blot analyses revealed that the treatment restored the expression of proteins associated with the cellular antioxidant response, including NRF2, HO-1, SOD2, CAT, and NQO1, suggesting reactivation of endogenous defense mechanisms against oxidative damage [78]. The system also demonstrated a significant protective effect on DNA, by monitoring biomarkers widely used to assess double-strand breaks and oxidative damage to genetic material, respectively [78]. These results indicate that nanozymes not only reduce ROS generation but also contribute to the maintenance of cellular homeostasis and DNA repair mechanisms [78]. The in vivo findings corroborated the in vitro results [78]. In an animal model of cisplatin-induced ototoxicity, treatment with this compound significantly reduced oxidative stress in the cochlear tissue, as evidenced by decreased levels of 4-hydroxynonenal (4-HNE), an important marker of lipid peroxidation [78]. Furthermore, preservation of auditory hair cells was observed, suggesting that the NAC-based strategy may represent a promising approach for preventing chemotherapy-associated hearing damage [78].

The antioxidant activity of hydrogels containing NAC has also been demonstrated in systems designed to protect against alcohol-induced gastric lesions [61]. In this context, a gastric acid-responsive hydrogel composed of chitosan functionalized with N-acetyl-L-cysteine (CS-NAC), sodium alginate, and tilapia peptide (TP) was developed for the treatment of gastric ulcers [61]. Considering that excessive alcohol consumption promotes intense oxidative stress through the excessive generation of reactive oxygen species, the authors evaluated the scavenging capacity of the material against different free radicals, including DPPH, superoxide anion (O_2_•^−^), and hydroxyl radicals (•OH). Although both TP and the CS-NAC/SA system exhibited antioxidant activity individually, the CS-NAC/SA/TP hydrogel exhibited significantly superior performance, with IC_50_ values of 2.20, 2.15, and 1.26 mg mL^−^ for DPPH, O_2_•^−^, and •OH, respectively. These results suggest that the combination of NAC and the peptide has a synergistic effect that enhances the elimination of free radicals, reinforcing the potential of these systems for controlling oxidative stress in inflammatory and injured environments [61].

Similarly, the incorporation of NAC into bioinks for 3D bioprinting has been proposed to minimize cell damage induced by mechanical extrusion forces during the printing process [93]. Evaluation of intracellular reactive oxygen species (ROS) load demonstrated that bioprinted MC3T3 cells exhibited ROS levels approximately 1.4 times higher than those observed in the control group (*p* < 0.01), indicating that the process promotes a significant accumulation of oxidative stress. In contrast, NAC incorporation resulted in a free radical scavenging activity of approximately 78%, reducing ROS levels to values comparable to those in the control group [93]. These results demonstrate that NAC can act as an effective cytoprotective agent in bioprinted systems, contributing to the maintenance of redox homeostasis 78% preservation of cell viability after construct fabrication [93].

### 6.2. Antimicrobial and Antibiofilm Activity

Microbial infections are one of the main challenges to the success of biomaterials applied in tissue regeneration and wound treatment, potentially compromising the repair process, prolonging inflammation, and promoting the development of chronic infections. This scenario is even more concerning when associated with the formation of microbial biofilms, inducing resistance against conventional antimicrobial agents. Therefore, the incorporation of bioactive compounds capable of preventing microbial colonization and reducing biofilm formation has been extensively investigated [10,25,31,32,35,37,39,46,47,64,71,73,89].

**Figure 10:**
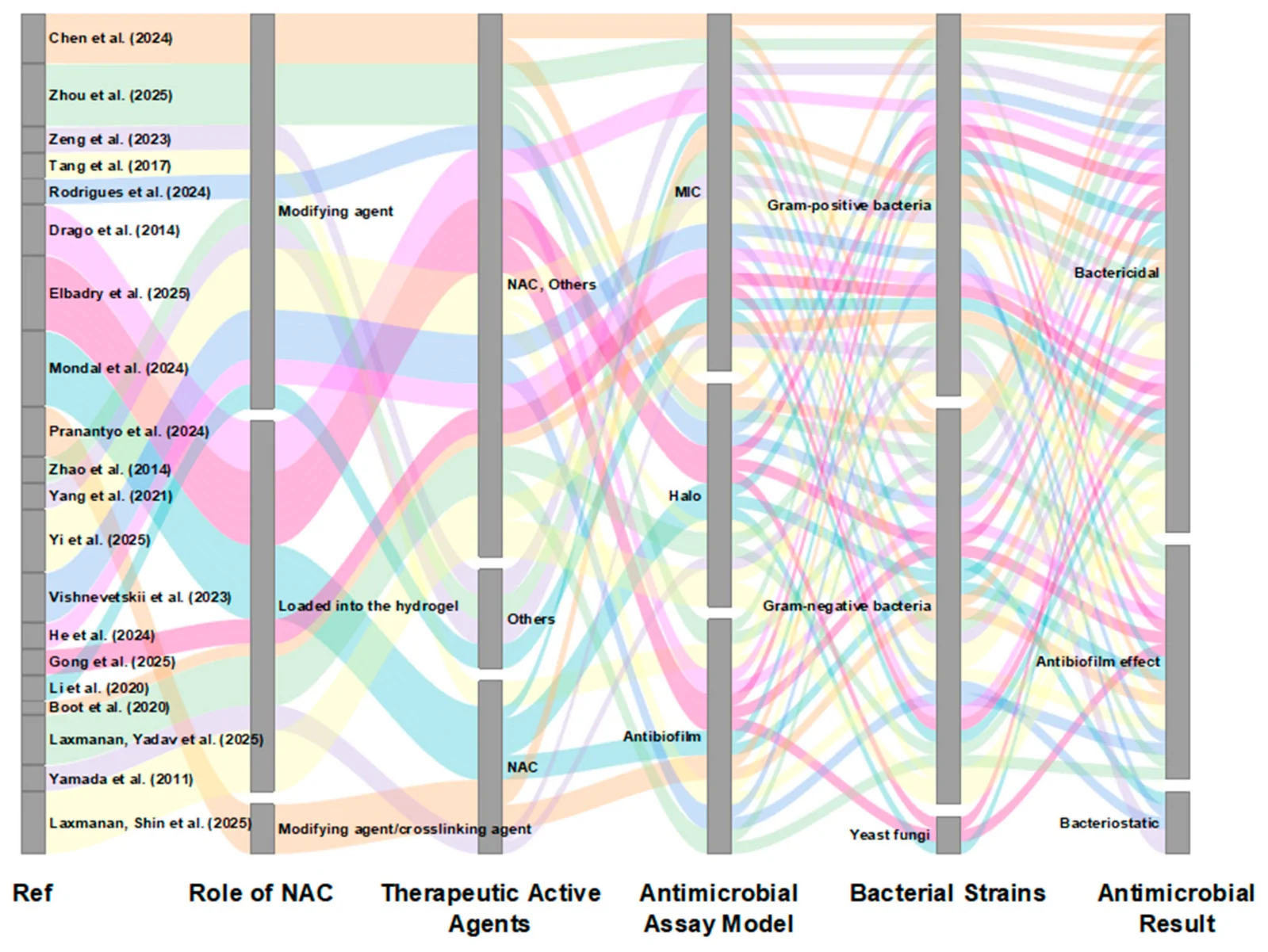
Alluvial diagram presenting the relationships among selected articles regarding NAC’s role into the hydrogel, therapeutic active agent, antimicrobial assay model, bacterial strains and antimicrobial result. The references cited in these diagrams are [10,25,31,32,35,37,39,41,42,44,46,47,50,64,67,68,71,86,88,89].

Although N-acetylcysteine is widely recognized for its antioxidant properties, recent studies have highlighted its promising antimicrobial and antibiofilm potential, which is often enhanced when combined with other active agents [59]. In this context, polymeric matrices such as chitosan and its derivatives can interact electrostatically with the bacterial cell wall, blocking nutrient transport, permeabilizing the membrane, and causing cell lysis [42]. Furthermore, incorporating therapeutic co-agents into the hydrogel’s three-dimensional network creates a synergistic effect that amplifies the system’s efficacy alongside NAC [42,87]. For instance, the presence of silver ions, nanoparticles, or nanowires disrupts the microbial envelope and halts protein synthesis and DNA replication through the sustained release of active ionic species (Ag^+^) [31,37]. Table 2 and Figure 10 below systematically summarize key literature findings, illustrating the broad biocidal and antibiofilm activity of NAC-hydrogels against Gram-positive and Gram-negative pathogens as well as yeast-like fungi.

From the 72 articles selected in the PRISMA flow diagram, only those that performed in vitro cellular studies were included in the Alluvial diagram (Figure 10), totalizing 20 studies. The height of the nodes represents the proportional distribution of these studies across the specified categories: the role of N-acetylcysteine (NAC), the therapeutic agents (NAC or NAC, other or other), because NAC was used in combination with other bioactive substances in several formulations (detailed in Table 2). The antimicrobial assay model, the bacterial strains and the antimicrobial results are also depicted.

Part of the studies combined NAC in conjunction with secondary therapeutic compounds, including vancomycin, Ag^+^ ions [67], polimidazoium, and tannic acid [88]. Additionally, in numerous studies, the intrinsic antimicrobial properties of chitosan also served as an active polymeric carrier. However, when NAC was evaluated as the sole therapeutic agent, it exhibited bacteriostatic [39,71], bactericidal [39,41], and antibiofilm activities [44].

Analysis of Figure 10 reinforces the multifunctional nature of N-acetylcysteine (NAC) within hydrogel. A review of the literature reveals that NAC’s role is primarily divided between the physicochemical modification of polymers and its direct delivery via the hydrogel. A strong correlation is observed between formulations combining NAC with other active agents and success in biofilm eradication assays and inhibition tests (MIC and halo assays). Consequently, these hybrid hydrogels exhibit predominantly bactericidal and antibiofilm behavior, suggesting that the combined presence of these compounds represents a strategy with excellent antimicrobial potential. As evidenced by the reported outcomes, this biocidal activity focuses primarily on combating Gram-positive and Gram-negative bacterial strains—given their significant clinical relevance—while also extending its therapeutic potential (albeit to a lesser extent in the analyzed reports) to yeast-like fungi.

Although N-acetylcysteine is traditionally recognized for its antioxidant and anti-inflammatory properties, studies have demonstrated its antimicrobial potential [71]. In this context, collagen scaffolds pre-treated with NAC were evaluated against strains of *Staphylococcus aureus 209P* and *Streptococcus pyogenes GTC 262* [71]. The results showed that NAC inhibited bacterial growth in a concentration-dependent manner, as observed by the reduction in turbidity of the cultures in BHI broth and the significant decrease in bacterial metabolic activity, evaluated through ATP levels [71]. Additionally, disk diffusion assays demonstrated the formation of inhibition halos, the extent of which increased with the concentration of NAC, confirming its antimicrobial activity [71]. However, subculture analyses performed from the inhibition zones revealed that the bacteria remained viable and recovered their growth capacity after the removal of NAC [71]. These results suggest that the molecule predominantly exerts a bacteriostatic effect, delaying bacterial proliferation without promoting complete elimination [71]. This property is particularly relevant for biomaterials intended for wound treatment and tissue regeneration, as reducing the microbial load helps minimize infections and promote the repair process [71].

**Figure 11:**
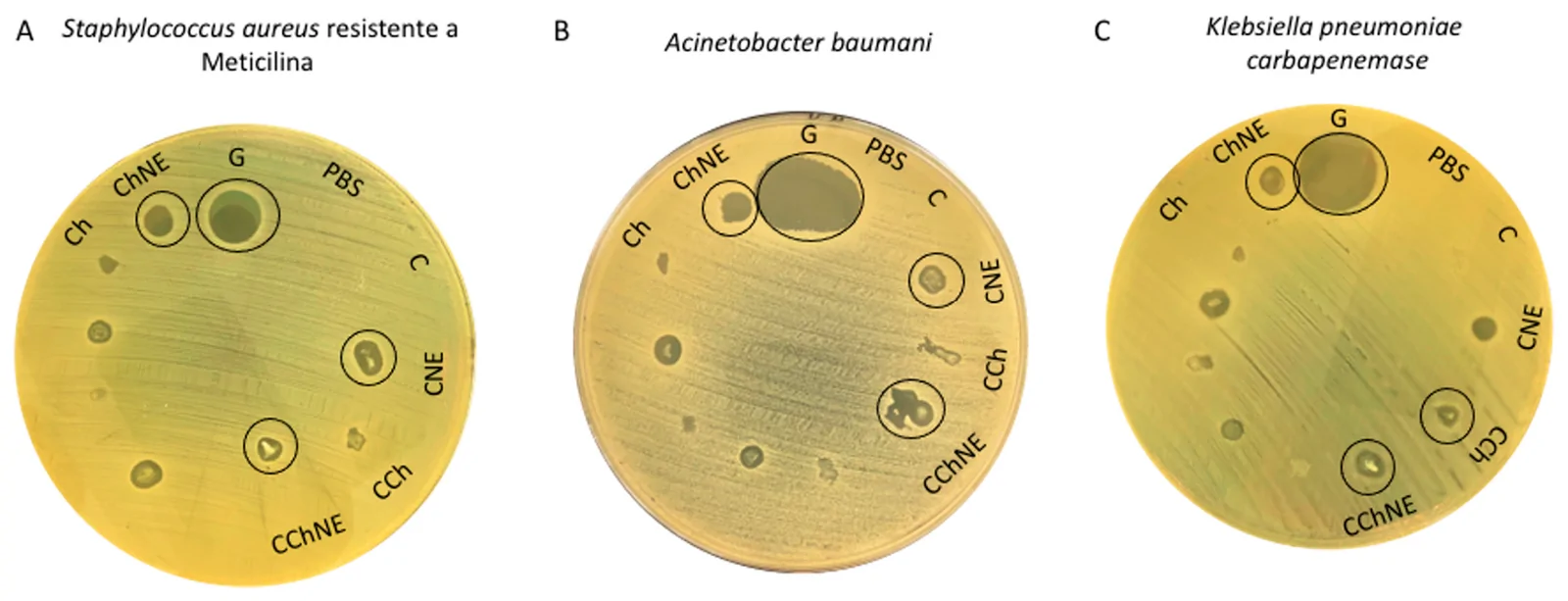
Antibacterial effect of scaffolds containing NAC–ε-PL. (**A**) Meticilin-resistant Staphylococcus aureus (**B**) Acinetobacter baumani (**C**) Klebsiella pneumoniae carbapenemase. Circles indicates the halo formation. G: Gentamicin; PBS: Phosphate-Buffered Saline; C: C: Collagen; CNE: Collagen–NAC–ε-PL; CCh: Collagen–Chitosan; CChNE: Collagen–Chitosan–NAC–ε-PL; Ch: Chitosan; ChNE Chitosan–NAC–ε-PL. This image is reproduced from [42]. under the terms of the Creative Commons Attribution License (https://creativecommons.org/licenses/by/4.0/ (accessed on 29 June 2026)).

On the other hand, Rodrigues et al. [42] developed scaffolds containing various combinations of collagen, chitosan, N-acetylcysteine (NAC), and ε-polylysine (ε-PL). Antimicrobial assays were qualitatively conducted using clinically relevant bacterial strains, including methicillin-resistant *Staphylococcus aureus* (MRSA), *Acinetobacter baumannii*, and carbapenemase-producing *Klebsiella pneumoniae* (KPC). The scaffolds containing NAC and ε-PL exhibited antibacterial activity against the tested strains (Figure 11), suggesting that these materials, in combination, may be promising for the development of biomaterials with antimicrobial potential [42].

NAC has also been investigated for its potential to prevent the formation of biofilms. In this context, Drago et al. [68] evaluated the antimicrobial and antibiofilm activity of a commercial biodegradable hydrogel (DAC^®^) loaded with different active agents, including NAC, against strains of *Staphylococcus aureus*, *Staphylococcus epidermidis*, *Escherichia coli*, *Enterococcus faecalis*, *Acinetobacter baumannii*, and *Pseudomonas aeruginos*. The minimum inhibitory concentration (MIC) results showed that the hydrogel alone did not exhibit any measurable antibacterial activity. However, when combined with antimicrobial agents, the system maintained or enhanced its effectiveness, with MIC values equivalent to or up to four times lower than those observed for the compounds tested in isolation. Additionally, hydrogels loaded with vancomycin, gentamicin, and NAC demonstrated a high capacity to reduce both initial bacterial colonization and the formation of new biofilms by *S. aureus* during incubation periods of up to seven days [68]. These results suggest that incorporating NAC into polymeric matrices can enhance the activity of other antimicrobial agents, contributing to the prevention of bacterial adhesion and biofilm establishment, which is particularly relevant in applications related to implants and biomaterials intended for tissue regeneration [68].

In addition to antioxidant activity, poly(methyl methacrylate) (PMMA) and marine collagen nanofibers loaded with NAC also showed significant antimicrobial and antibiofilm activities [89]. The material was evaluated against different microorganisms, including *Staphylococcus epidermidis*, *Staphylococcus aureus*, *Bacillus cereus*, *Salmonella paratyphi*, *Escherichia coli*, *Klebsiella pneumoniae*, *Candida glabrata*, *Candida albicans*, and *Candida parapsilosis*. In general, all formulations demonstrated antimicrobial activity against *B. cereus*, *S. paratyphi*, *E. coli*, and *K. pneumoniae*, with the formulation containing 10% NAC (3PMMA:1MC/10%NAC) showing the largest inhibition halos for most of the strains evaluated. In addition to its effect on planktonic microorganisms, the optimized formulation exhibited a high capacity to inhibit biofilm formation promoting reduction greater than 90% for most of the pathogens tested. This performance was significantly superior to that of conventional antibiotics, such as kanamycin, chloramphenicol, and tetracycline. The results suggest that incorporating NAC into fibrous systems contribute not only to reducing microbial growth but also to controlling biofilm formation, which is one of the main factors associated with persistent infections and therapeutic failures in biomaterials [89].

### 6.3. Tissue Regeneration and Wound Healing

Tissue regeneration and wound healing are complex processes that involve the coordination of multiple biological events, including cell migration, proliferation, angiogenesis, extracellular matrix remodeling, and modulation of the inflammatory response. Thus, biomaterials containing N-acetylcysteine (NAC) have attracted interest because of their ability to simultaneously modulate different factors involved in healing. In addition to reducing oxidative stress and attenuating exacerbated inflammatory responses, these systems have shown potential to stimulate cell migration and proliferation, promote the formation of new blood vessels, and accelerate wound healing in various experimental models [27,95]. Table 3 summarizes the main in vivo results extracted from the articles considered in the systematic review, detailing the animal models, experimental assay, hydrogel types and primary outcomes.

**Figure 12:**
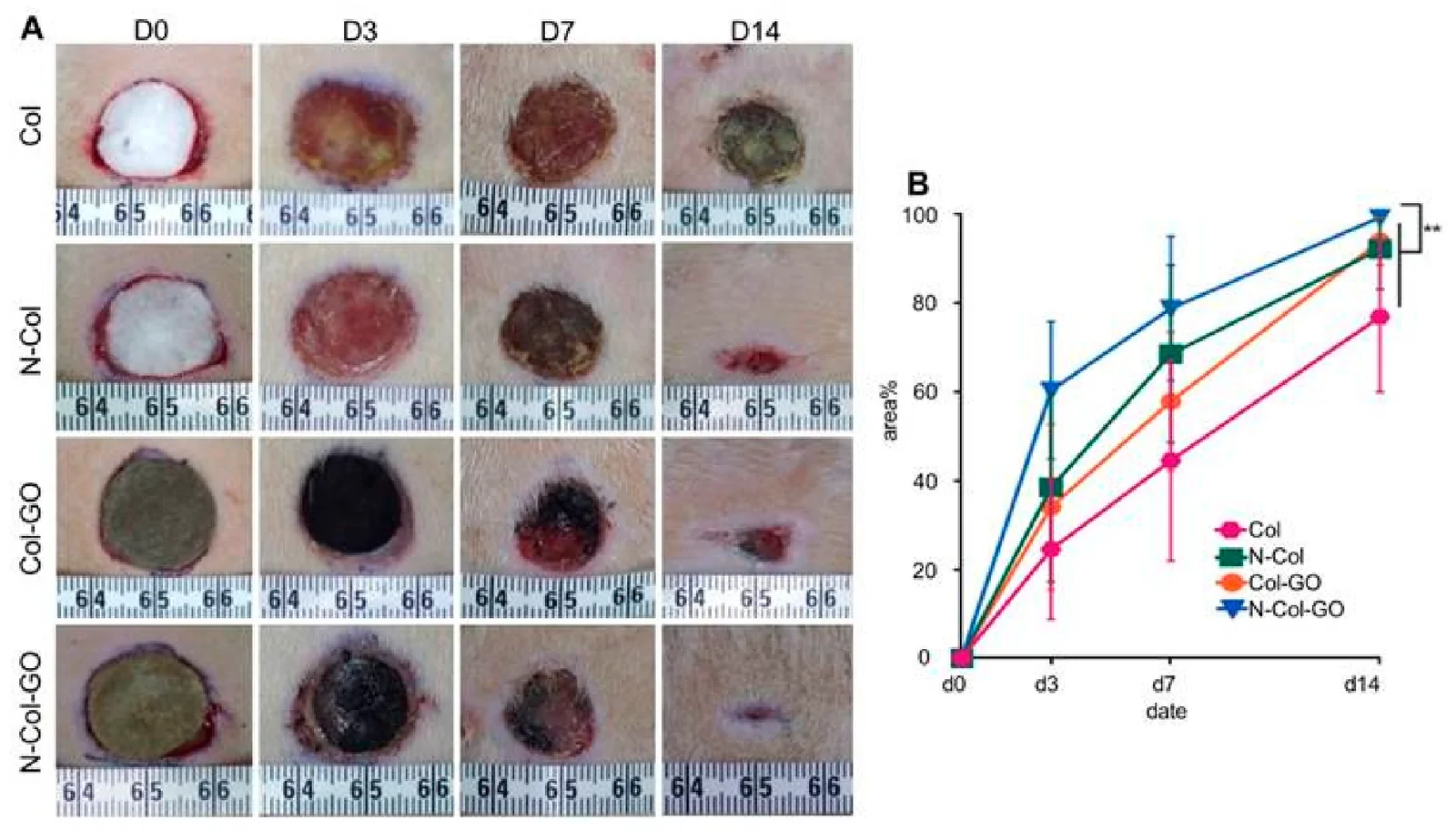
20 mm dermal defect repaired by Col, Col, N-Col, Col-GO, and N-Col-GO hybrid membranes in rats. (**A**) Photographic evaluation of skin regeneration on day 14. N-Col-GO has completely healed while other groups failed. (**B**) Area ratio of rat wound healing (** *p* < 0.01). This image is reproduced from [36] under the terms of the Creative Commons Attribution License (https://creativecommons.org/licenses/by/4.0/ (accessed on 29 June 2026)).

In a composite based on collagen, graphene oxide (GO), and NAC, the incorporation of the antioxidant played a key role in reducing the cytotoxicity associated with GO, favoring the interaction between the biomaterial and the cells. In vitro assays using NIH 3T3 fibroblasts, the groups containing NAC (N-Col-GO) showed greater cell viability and faster migration when compared to the other groups. The in vivo results corroborate these findings. Figure 12 shows that skin wounds treated with N-Col-GO showed complete healing after 14 days, accompanied by total re-epithelialization of the tissue. Additionally, the composite containing NAC promoted an approximately twofold increase in the number of blood capillaries in the injured area, indicating angiogenesis stimulation. Taken together, these results demonstrate that the incorporation of NAC can favor different stages of the healing process, including cell migration, neovascularization, and tissue regeneration [36].

Adalghi et al. developed a hydrogel based on carboxymethylcellulose, gelatin, and sodium alginate (CMC/Gel/Alg) incorporating N-acetylcysteine for the treatment of pressure ulcers. In an in vivo model, the hydrogel containing 5 mg mL^−1^ of NAC promoted 84.32% wound closure after 14 days of treatment, a performance superior to that observed in the control group, the hydrogel without NAC, and the commercial product ChitoHeal Gel^®^. Histological analyses revealed complete re-epithelialization and adequate regeneration of the epidermis, dermis, sebaceous glands, and hair follicles, indicating advanced structural recovery of the injured tissues. Additionally, immunohistochemistry demonstrated a reduction in TNF-α expression, suggesting the modulation of the local inflammatory response. Gene expression analysis corroborated these results, showing increased levels of type I collagen and TGF-β1, associated with the formation and remodeling of the extracellular matrix, accompanied by a reduction in the expression of MMP2 and MMP9, which are enzymes involved in tissue degradation. Taken together, these findings demonstrate that the incorporation of NAC favors multiple stages of the healing process, from inflammation control to tissue regeneration and remodeling [62].

Zhou et al. [64] developed a hydrogel composed of acrylated aspartic acid (AASP), carboxymethylated chitosan grafted with N-acetylcysteine (CMCS-NAC), and amphiphilic molecules responsive to gastric pH (C_16_N-DCA), aimed at treating gastric mucosal lesions [64]. The material showed rapid adhesion (5 to 22 s) to the injured tissue by forming a physical barrier capable of reducing pepsin infiltration by approximately 93% in vitro and promoting wound sealing in vivo. In an endoscopically treated porcine model of induced gastric ulcer, the hydrogel maintained strong adhesion to the injured mucosa for at least 12 h, even under intense gastric motility, continuous fluid flow, and acidic conditions. Furthermore, histological analyses did not show significant infiltration of inflammatory cells, demonstrating the adequate biocompatibility and safety of the material [64]. The therapeutic efficacy of the system was evaluated in a murine model of chronic skin wounds infected with Staphylococcus aureus. The treatment significantly accelerated the healing process, resulting in approximately 97% wound closure after 14 days, a value substantially higher than that observed in the control groups. Histological and immunohistochemical analyses demonstrated a reduction in TNF-α expression, an increase in the angiogenic markers VEGF and CD31, more organized collagen deposition, and accelerated re-epithelialization. Taken together, these results indicate that hydrogels favor multiple events involved in tissue regeneration, including inflammation control, neovascularization, and extracellular matrix remodeling [64].

The application of biomaterials containing NAC has also been explored for the regeneration of complex abdominal wall defects. In this context, Liu et al. [38] developed a bilayer patch composed of polycaprolactone (PCL) nanofibers, chitosan (CS), and graphene oxide (GO), functionalized with NAC in the inner layer, to promote tissue reconstruction and prevent postoperative adhesions. In a rat model of total abdominal wall defects, the incorporation of GO with and without conferred sufficient mechanical resistance to prevent hernia formation, as no animal treated with GO-containing materials developed this complication. Additionally, the presence of the chitosan layer promoted a significant anti-adherent effect, resulting in adhesion rates significantly lower than those observed in the control group. Histological analyses demonstrated adequate integration of the biomaterial into the host tissues, accompanied by the formation of new connective tissue in the repaired area. Immunohistochemical evaluation revealed the presence of newly formed blood vessels predominantly in the NAC-containing group, suggesting stimulation of local angiogenesis [38]. Furthermore, this group showed denser new tissue and the highest collagen deposition among the materials evaluated (34.94 ± 3.31%), with a predominance of type I collagen, indicative of a more mature and mechanically stable tissue, Figure 12. Overall, the results demonstrate that the incorporation of NAC contributes to the creation of a microenvironment favorable for tissue regeneration [38].

**Figure 13:**
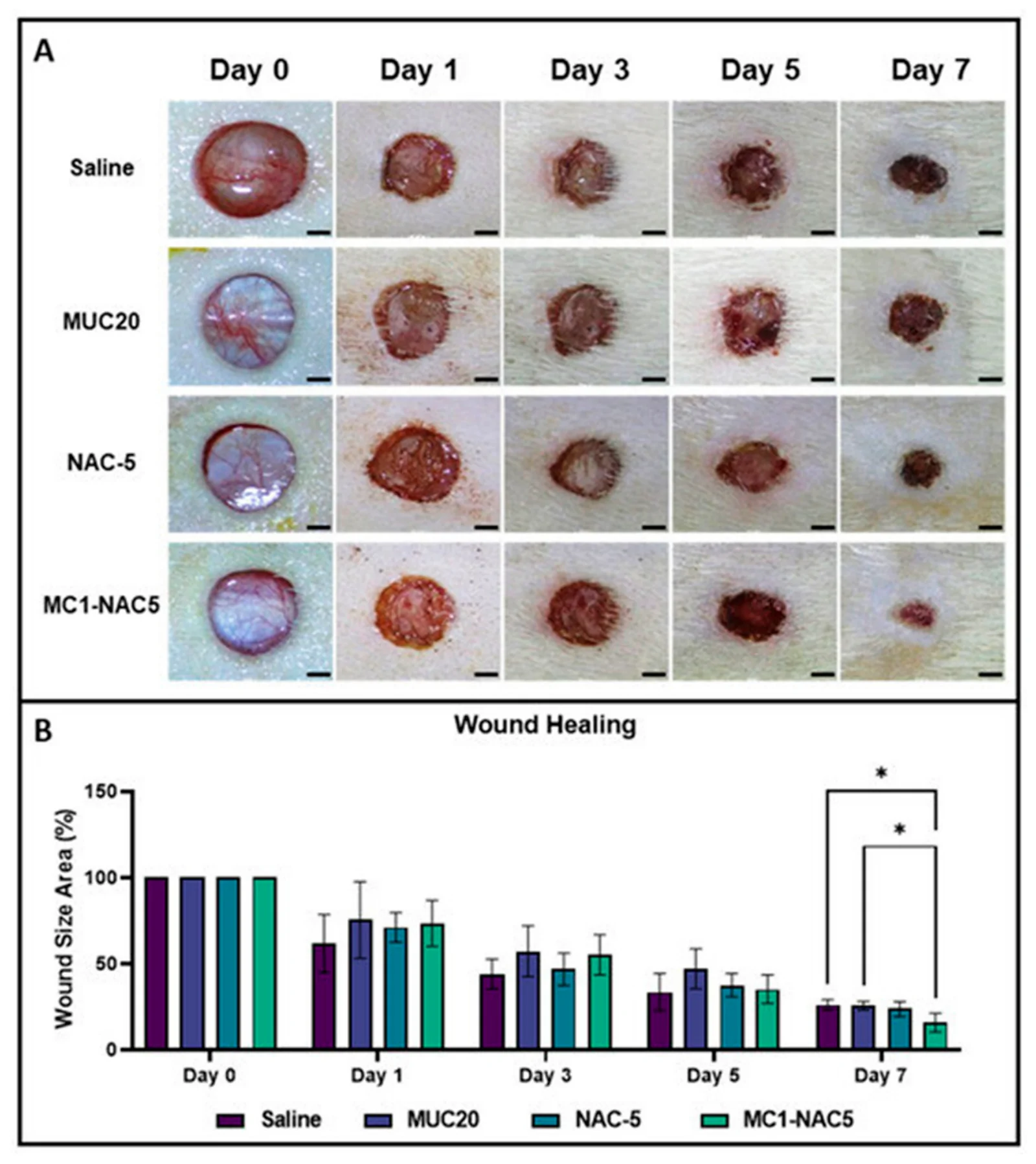
Macro images of the wound beds routinely treated with either saline, MUC20, NAC-5, or MC1-NAC5 (**A**) (scale bar = 2 mm). Results of the wound size analysis (**B**) indicate that application of MC1-NAC5 resulted in a significantly reduced wound size at day 7 but did not show any significant reduction within the 1-week observation period (*n* = 4, * *p* < 0.05). This image is reproduced from [16] under the terms of the Creative Commons Attribution License (https://creativecommons.org/licenses/by/4.0/ (accessed on 29 June 2026)).

To increase the residence time of NAC at the injury site, Kim et al. developed a thermosensitive hydrogel composed of methylcellulose (MC) and N-acetylcysteine (NAC) for the treatment of dermal wounds and oral ulcers. The formulation containing 1% MC and 5% NAC demonstrated the best therapeutic performance, promoting an approximately 85% reduction in the dermal wound area after seven days of treatment, which was superior to that observed with free NAC, methylcellulose alone, or the control group, as shown in Figure 13. In an oral ulcer model, the hydrogel also significantly accelerated healing, promoting near-complete mucosal regeneration, whereas NAC administered as a solution showed only a modest effect. Furthermore, histological analyses corroborated these findings, demonstrating the formation of a thicker and better-organized epithelium in lesions treated with hydrogel. Immunohistochemical evaluation revealed increased MMP-2 expression, suggesting intense tissue remodeling and cell migration, along with a significant reduction in the pro-inflammatory cytokines IL-6 and TNF-α. The authors attributed these results to the increased viscosity provided by methylcellulose, which facilitated the local retention of NAC and prolonged its therapeutic action [16].

Thus, compiled data demonstrate that incorporating N-acetylcysteine (NAC) into polymeric matrices extends beyond conventional cutaneous wound repair, positioning it as a potential therapeutic platform with broad applicability in regenerative medicine and tissue engineering [15,16,42]. Due to its chemical versatility, NAC can function either passively, through physical encapsulation or dispersion for controlled release, or actively, acting as a structural modification agent that alters polymer networks to optimize properties such as mechanical strength, swelling rates, and biological stability [16,17,38,49]. Consequently, its structural flexibility enables application in highly specialized tissues, ranging from providing mechanical support and biological stimulation to developing photo-responsive and bioadhesive systems for healing gastric mucosal lesions and regulating hemostasis [17,49,63,64,69,76,93]. Furthermore, NAC’s neuroprotective and antioxidant capabilities significantly expand its utility to the fields of peripheral nerve regeneration and intranasal delivery systems targeting brain tissue, where controlling oxidative stress and restoring cell viability are critical for therapeutic success [52]. Overall, the various scenarios demonstrated in these studies indicate that NAC-containing materials stand out as potential therapeutic solutions capable of dynamically responding to the biological and physical requirements of diverse organs and systems. Topical NAC hydrogels, films, nanofibrous matrices, and particulate systems may improve stability, prolong skin residence, and maintain NAC within superficial tissue compartments [29,39,89]. Such retention may maximize local antioxidant activity while limiting systemic exposure. These properties support potential applications in barrier protection, photodamage prevention, post-procedural recovery, and anti-aging formulations. Nevertheless, cosmeceutical evidence remains limited, requiring further evaluation of dermal tolerance, repeated-use safety, skin deposition, storage stability, packaging compatibility, and comparative efficacy against established topical antioxidants.

## 7. Conclusions

It is important to highlight that NAC is a long-standing active pharmaceutical ingredient documented in major official compendia, such as the European Pharmacopoeia [20]. Its well-defined physicochemical, analytical, and pharmacological profiles strongly endorse the rationale for engineering advanced hydrogel delivery platforms.

Overall, the application of NAC in hydrogel systems has shown promising results, promoting responsive behavior and improving tissue regeneration, adhesiveness, resistance, and anti-inflammatory response.

Although extensive efforts have been made to achieve sustained drug release, most formulations still exhibit a pronounced initial burst effect, highlighting that achieving fully controlled release remains a critical challenge. To overcome this limitation, a deeper understanding of how NAC-induced chemical modifications of hydrogel networks is essential. In this context, advanced characterization techniques, such as XPS, NMR, and Raman spectroscopy, are strongly recommended to elucidate the precise structural modifications within the polymer matrix.

The challenges encountered when correlating data across various studies clearly demonstrate the need for standardized analytical methodologies to quantify NAC and its degradation product. To enhance the analytical reliability of NAC quantification, future studies should establish limits of detection (LOD) and quantification (LOQ). These parameters are critical for defining sensitivity thresholds, particularly in skin permeation assays where analyte concentrations may fall below quantifiable levels. Additionally, potential analytical artifacts, such as NAC oxidation or degradation during release studies, incomplete recovery, and non-specific adsorption onto experimental apparatus, must be addressed. Matrix recovery assays are also required to ensure quantitative extraction from the polymer network, thereby guaranteeing an accurate mass balance and comprehensive tracking of NAC distribution across the polymer matrix, receptor fluids, and skin tissue.

In summary, the demonstrated in vitro and in vivo wound-healing and antioxidant capabilities of NAC-containing hydrogels, combined with NAC’s established dermatological benefits [97], highlight their cosmeceutical potential. Nevertheless, randomized controlled clinical trials are required to validate their efficacy in clinical practice.

Although NAC-loaded hydrogels show high potential as biomedical devices, translational challenges remain. Future research should focus on optimizing scalable production to ensure reproducibility. Additionally, comprehensive stability testing is required to evaluate shelf life, degradation kinetics, and sustained antioxidant activity under standard storage conditions. Finally, addressing regulatory standards for sterility and biocompatibility will be essential to advance these hydrogel systems toward commercialization.

Overall, the analyzed articles demonstrate that NAC plays multifunctional roles in polymeric hydrogels due to its chemical structure, yielding gels with varied physicochemical properties depending on the polymers, crosslinking agents, and metallic ions utilized. While this structural diversity significantly expands the range of tailorable hydrogel characteristics, it inherently increases the complexity of conventional design approaches. Consequently, innovative strategies are required to mitigate high experimental costs and time-consuming trials. In this context, recent studies highlight the potential of artificial intelligence (AI) in hydrogel design and optimization, enabling property prediction and efficient experimental planning. Therefore, AI-driven approaches represent a promising trend for future research on NAC-containing hydrogels [98,99].

Notably, the NAC molecule can modify polymeric structures to impart pH-responsiveness [46,100], which is a key feature in the development of smart hydrogels. However, literature directly exploring this specific capability of NAC remains scarce.

Hydrogels are essential components in 3D bioprinting, serving as bio-inks to fabricate complex tissue structures, encapsulate viable cells, and provide a supportive microenvironment for cell proliferation and differentiation. Furthermore, these systems can sustain the delivery of growth factors, antioxidants, and crosslinking agents—such as the multifunctional NAC molecule [101]. Consequently, incorporating NAC into bio-inks represents a highly promising research topic for developing multifunctional biomaterials and advancing personalized healthcare.

Finally, the literature compiled in this systematic review indicates that the translational process of NAC-based hydrogels from laboratory discovery to clinical care remains in an early stage. Although select studies demonstrate therapeutic efficacy in preclinical disease models, particularly for wound healing applications, the vast majority remain restricted to basic research. Bridging this gap requires collaborative efforts from interdisciplinary teams to establish a robust clinical translation roadmap. To achieve this objective, it is necessary to overcome some key technical and biological challenges, notably the implementation of validated analytical assay methods to evaluate NAC stability and shelf life, strict adherence to regulatory standards for biological evaluation, and strategic cross-sector partnerships between academia and industry to advance in human studies and clinical trials.

## Figures and Tables

**Table 1:** Summary of drug release assay reported by the studies within the scope of this review.

Ref	Therapeutic Substance	Release Assay (Medium, Time, Quantification Technique)	Swelling Degree	EE	Cumulative Release Behavior	Role of NAC/ Application
[65]	Atropine	PBS with physiologic concentrations of lysozyme, 120 h; HPLC	72–76% in 24 h. (NaCl 1 M)	N.P.	The atropine release was almost complete over 8 h, and stabilized after 24 h on 89% to the CTS-pNAMP-NAC3 hydrogel.	Modifying agent/crosslinking /ocular treatment
[66]	Avastin, Dexamethasone	PBS pH 7.4, 216 h (9 d); ELISA (UV-Vis)	N.P.	68.01 ± 3.24%	The authors compared Avastin loaded in hydrogel (PF127 and HPMC) and encapsulated onto CS-NAC nanoparticles that are incorporated in hydrogel. The release of free Avastin carried on the hydrogel was much faster than the release of Avastin encapsulated. In 24 h, the free Avastin was released almost completely, while around 10% was released from the Avastin encapsulated. 97% of dexamethasone, free in the hydrogel, was released in 18 h.	Modifying agent/ocular treatment
[74]	Gemcitabine (GEM) and Celecoxib (CEX)	PBS pH 6.8, 37 °C, HPLC	Not determined		GEM and CEX by continuing release over time from NAC-glycol-chitosan hydrogel, achieving 15% and 90%, respectively over 6 h, without bust effect. Kinetics analysis suggests that hydrogel was not subjected to major swelling or erosion. Due to strong interaction of the gel with pig bladder mucosa, zero percentage of CEX and 7.60% of GEM was permeated.	Modifying agent/mucoadhesive
[64]	C_16_N^+^ particles	PBS pH 7.4 and SGF pH 2.0;	Around 160% (PBS pH 7.4) and 120% (SGF pH 2.0),	N.P.	The particle release is slow, with an initial release of 42.63% within 10 h. Maximum release (92.36%) occurred at 36 h from CMCS-NAC-AASP hydrogel.	Modifying agent/gastric mucosa lesion
[54]	Egg white-derived peptides (EWPD) and curcumin	SIF and SGF, 58–70 h RP-HPLC-UV and multimode microplate reader.	N.P.	51–89% (EWPD, pH 2–7.4) 41–57% (curcumin, pH 2–7.4)	A burst release of EWPD and curcumin in the first 2 h in SGF was shown from CS-NAC-β-lg hydrogels, around 10%. The following time was performed in SIF; the release of EWPD was 43% in 10 h. It is followed by a gradual release, reaching 66% at 58 h. Curcumin release was 17% in 10 h and scaled to 51% in 70 h. The release of EWPD and curcumin is inhibited at lower pH. The authors reported that curcumin release by the NAC- or CYS-modified chitosan is slower when compared to the literature of curcumin entrapped in other chitosan-based hydrogels.	Modifying agent/drug delivery
[58]	Insulin and Bovine Albumin (BSA)	PBS pH 7.4, 72 h; In vitro, Bradford assay and SDS-PAGE	N.I.	N.P.	An initial burst effect is followed by slower release attributed to protein–chitosan interaction. The disulfide bonds promoted by NAC affected the insulin content released, enhancing from 58.4% to 61.3% in 72 h with the increase in disulfide content, and consequently, crosslinked density.	Modifying agent/crosslinking
[52]	NAC	PBS pH 7.4, 6 h; In vitro, UV-Vis spectrophotometry	N.P.	N.P.	56.5 ± 1.5% (free NIR), 65 ± 0.5% (NIR application) from DA-HA–silk fibroin–BDDE hydrogel.	Loaded into the hydrogel/drug delivery
[62]	NAC	SBF, 16 h; UV-Vis spectrophotometry	226.96% after 12 h.	N.P.	NAC released during the first 6 h was 18.12 ± 1.76%. Subsequently, a sustained release of 97.55 ± 2.45% was observed over the following 16 h, and it did not reach full release from CMC/gelatin/alginate hydrogel.	Loaded into the hydrogel/ulcer treatment
[89]	NAC	PBS, 6 h; UV-Vis spectrophotometry	N.R. Around 1700%	N.P.	Cumulative release curve is not presented. The release was evaluated qualitatively from PMMA/Col hydrogel.	Loaded into the hydrogel/topical delivery
[39]	NAC	SWF, 24 h; Modified Franz diffusion cells, ex vivo goat abdomen tissue, UV-Vis spectrophotometry	From 105 ± 2.82% to 320 ± 0.70% after 1 h, depending on the formulation.	From 82.5 ± 2.54 to 97.68 ± 0.26	In vitro release results had different behavior according to the formulation (Alginate/guar gum). The best result (formulation F3, 92.538 ± 3.89%) displayed a sustained release over 8 h. The kinetics of release (non-Fickian) indicate the release is modulated via swelling followed by erosion and diffusion. In vitro permeation assay displayed a nonlinear curve; a rapid release of 17% occurred in 1 h. Over 24 h, the amount of NAC permeated was 103.53 ± 1.80%. C_max_ Skin: 86.12 ± 3.123 μg/cm^2^ (epidermis) and 67.78 ± 3.657 μg/cm^2^ (dermis); T_max_: 5.00 h (epidermis) and 9.00 h (dermis).	Loaded into the hydrogel/Diabetic wound
[15]	NAC	PBS pH 7.4, 145 min; ABTS^•+^ assay, UV-Vis spectrophotometry	1200–1400%	N.P.	A fast release of more than 60% of NAC was observed after 15 min, followed by the slowest and nearly complete release of over 145 min from the Gel-HP-NAC hydrogel.	Loaded into the hydrogel/wound healing
[77]	NAC	PBS pH 7.4, 16 days; UV-Vis spectrophotometry	N.R.	N.P.	The first detectable release of NAC began 5 h after application, and the release rate remained constant for the first 24 h. After about 5 days (132 h), the release level increased to 60%. By the end of 16 days, the hydrogel had released 86.3% ± 11.4% of the total NAC loaded in CS/PVA hydrogel.	Loaded into the hydrogel/hearing loss prevention
[26]	NAC	PBS at 37 °C, HPLC	600 to 7000% in water, depending on the composition		Crosslinked GO-Col-NAC using EDC/NHS promotes sustained NAC release for 18 days, 51% in 24 h, and 72% cumulative release up to 18 days.	NAC as antioxidant/ diabetic wound dressing
[27]	NAC	Buffer pH 7.4 or 5.5 in 2% (*w*/*v*) at 37 °C, HPLC	334% in 2 h		Hydrogel of chitosan bonded to CL-LA-PEG200-PEA copolymer released 71 to 86% of NAC (24 h) and 100% at 50–55 h, governed by non-Fickian transport model.	NAC as antioxidant/diabetic wound dressing
[33]	NAC	PBS at 37 °C, UV-vis	370% in water		Scaffold of PCL nanofibers recovered by Col/NAC crosslinked with EDC-NHS present ca 60% (24 h) and 65% of cumulative release after 14 days, classified as sustained release.	NAC as antioxidant/diabetic wound dressing
[87]	NAC and bFGF	PBS and PBS-H_2_O_2_ pH 6.5 and 7.4; 72 h; FITC (fluorescence), HPLC	N.R.	N.P.	NAC release in oxidative media (PBS + H_2_O_2_) at pH 6.5 was much higher than in PBS, reaching nearly 93.4% in 48 h. The bFGF release was slower; it reached 66.5% after 72 h, without achieving full release. Release was modulated by the S-S bond cleavage of a NaHA-PDA-PEG hydrogel.	Modifying agent/wound healing
[29]	NAC and Lysine (LYS)	PBS pH 5.5, 6 h (release), 24 h (permeation); Franz diffusion cells; HPLC	N.P.	N.P.	N.R. The release of NAC and Lys from the Carbopol hydrogel followed the Higuchi model, indicating a controlled and Fickian release behavior. There was a dose depletion of approximately 17% for each molecule. No significant quantities of NAC or Lys were permeated through skin, the active compounds remaining on the skin surface.	Loaded into the hydrogel/wound healing
[82]	Nitric oxide from NAC bond on CS	Photometric	600 to 1100% in PBS, depending on the composition in PBS buffer	N.P.	Crosslinked CS/HA-NH_2_ anchored with NAC as S-nitrosothiol group bonds the sustained release of NO during 46 h (72 nmol/mg).	Modifying agent, NAC as NO donor/wound dressing
[49]	Pro-angiogenic peptide derived from adrenomedullin (PAMP) and NAC	PBS pH 7.4, 14 d; Ninhydrin assay, UV-Vis spectrophotometry	~1.24 (NAC), ~1.16 (PAMP and NAC), ~1.08 (PAMP), >1.08 (without drug carried out).	N.P.	NAC and PAMP were loaded into GelMA and ChMA hydrogels, separated and combined. NAC-only hydrogel showed an initial burst release within the first 48 h. The hydrogel containing only PAMP showed the lowest amount of drug released. The co-release displayed an intermediary cumulative release at 48 h. In 7 days, the drug release for all samples was around 70–80%.	Loaded into the hydrogel/ osteoregeneration
[70]	Sr^2+^ and NAC	Trizma, 14 d; HPLC and ICP-AES	4.6% (pH 7.4) after 1 day	N.P.	The NAC release from bioactive glass/poly(ether urethane) hydrogel (MBG/SHP) was much slower when the particles were incorporated in the hydrogel. The NAC released in the first 3 h was around 20%, and 60% after 24 h (5 mM), it took 7 days to reach 90% (8 mM) of NAC released. In the particles, all of NAC content was released in the first hour. 56% of Sr^2+^ ions were released after 7 days.	Loaded onto the nanoparticles/ osteoregeneration
[63]	Tilapia collagen peptide (TCP)	SIF and SGF pH 6.8, 6 h; In vitro, UV-Vis spectrophotometry	N.P.	N.P.	A burst release of TCP was shown in the first hour in SIF and SGF. After 6 h, the release reached 95.20% (SGF) and 98.39% (SIF).	Modifying agent/ protection on mucosa
[50]	Vancomycin	PBS pH 7.4; 48 h; UV-Vis spectrophotometry	250% (on average) in 1, depending on the formulation	N.P.	The cumulative vancomycin-loaded release strongly depends on the formulation. The higher the thiol/maleic acid and the higher the dextran/CS-NAC content, the lower was the drug release. This can be a result of interaction of Vancomycin with dextran due to crosslink density. The release mechanism followed a non-Fickian diffusion to the formulation with the higher and the lowest dextran content with sustained release after 50 h (ca. 50%).	Modifying agent/ multipurpose application
[68]	Vancomycin, Gentamicin, Amikacin, Tobramycin, Sodium salicylate, NAC	FCS and HS, 96 h; In vitro, Photometric measurement	N.P.	N.P.	Rapid release from commercial gel, with maximum release between 2–4 h. Complete or nearly complete release to all drugs tested in less than 96 h.	Loaded into the hydrogel/ osteoregeneration

Acronyms: N.R.—No quantitative percentage is reported in the text; PBS—phosphate-buffered solution; N.P.—Not performed; CTS-pNAMP-NAC3—(chitosan-poly(n-isopropylacrylamide-co-acrylic acid-co-methyl methacrylate-co-pyridyl disulfide ethylmethacrylate)-acetylcysteine); HPLC—high-performance liquid chromatography; SDS-PAGE—Sodium dodecyl sulfate polyacrylamide gel electrophoresis; FCS—fetal calf serum; HS—human serum; NIR—near-infrared irradiation; FITC—fluorescein isothiocyanate isomer; SBF—simulated body fluid; SWF—simulated wound fluid; SIF—simulated intestinal fluid; SGF—simulated gastric fluid; ICP-AES—inductively, coupled plasma atomic emission spectroscopy; RP-HPLC—reverse phase high-performance liquid chromatography.

**Table 2:** Summary of antimicrobial assays reported by the studies included in this review.

Ref	Role of NAC	Antimicrobial Effect/MIC	Antibiofilm Effect
[89]	Loaded into the hydrogel	Zones of inhibition: 34.37 mm (*S. aureus*), 33.89 mm (*C. glabrata*), 31.24 mm (*K. pneumoniae*).	96.46% reduction (*S. epidermidis* at 8 h) and 97.18% (*C. glabrata* at 12 h).
[39]	Loaded into the hydrogel	Zones of inhibition: 1.9 cm (*S. aureus*), 2.16 cm (*E. coli*), 2.3 cm (*C. albicans*). Cell permeability at an MIC of 50 µg/mL.	Biofilm inhibition of S. aureus (28.29%) and *E. coli* (43.62%) at 12.5 µg/mL.
[86]	Modifying agent	MIC of 46.9 µM for Gram-positive and 11.7 µM for Gram-negative bacteria (SS-7 system).	Complete biofilm inhibition of *A. baumannii* and *P. aeruginosa* (20–40 µM).
[25]	Modifying agent	Thiolated chitosan (without laser): 45% (*S. aureus*) and 23% (*E. coli*). With NIR laser: 100% elimination.	Not evaluated.
[32]	Loaded into the hydrogel	Membrane destroyed by lysis. In infected rats: only 55 CFU/mL of *S. aureus* remaining on day 3.	Not evaluated.
[67]	Loaded into the hydrogel	Ineffective in vivo against tibia infection; bacterial load of 6.6 × 10^6^ CFU/g.	Ineffective in preventing biofilm in vivo.
[88]	Loaded into the hydrogel	Zones of inhibition: 28 mm (*E. coli*) and 29.5 mm (*S. aureus*) (NAPT formulation).	NAPT strongly inhibited *S. aureus* biofilm formation as evaluated by crystal violet.
[71]	Loaded into the hydrogel	Dose-dependent bacteriostatic action against *S. aureus* and *S. pyogenes* in BHI broth for 12 h.	More than 15-fold reduction in *S. aureus* invasion of gingival fibroblasts.
[35]	Loaded into the hydrogel	Under 808 nm NIR irradiation: log10 reduction of 4.28 (*E. coli*) and 3.19 (*S. aureus*) by photothermal ablation.	Photothermal eradication of bacterial biofilms.
[42]	Modifying agent	Formation of clear zones of inhibition against clinical multidrug-resistant bacteria: MRSA, Acinetobacter baumannii, and KPC.	Prevents the formation of new biofilms by resistant pathogens.
[68]	Loaded into the hydrogel	Reduced in vitro MIC up to 4-fold in association with hydrogel (to 6.125 mg/mL against *S. epidermidis*, *S. aureus*, etc.).	Significant reduction in mature *S. aureus* and *S. epidermidis* biofilm on titanium, polyethylene, and cobalt-chromium from 2–4 h up to 48 h.
[31]	Modifying agent	Achieved an elimination rate greater than 99% against *E. coli* and *S. aureus* in vitro due to the controlled release of silver ions.	Not evaluated.
[10]	Modifying agent	Sustained release of NAC resulted in significant bactericidal activity against *E. coli* and *S. aureus* in vitro.	Antimicrobial activity via destruction of the bacterial redox balance inhibits the formation of new biofilms in burns.
[64]	Modifying agent	Under simulated acidic stomach pH (pH 3), it underwent charge reversal, eliminating Helicobacter pylori with 98% efficacy in vitro for up to 36 h; potently eliminated *E. coli* and *S. aureus* in vitro.	Strongly inhibited the formation and viability of mature *E. coli* and *H. pylori* biofilm.
[44]	Modifying agent	Zwitterionic hydrogel CS1DM3 prevented bacterial adhesion of *S. aureus* and *E. coli* in 12 h assays.	Prevented initial biofilm formation through anti-adhesion (antifouling) properties.

**Table 3:** Main in vivo results extracted from the articles considered in this systematic review, detailing the animal models, experimental assay, hydrogel types and primary outcomes.

Ref	Animal Model	Assay Description	Evaluated Hydrogel	Main In Vivo Result
[59]	C57BL/6 mice	Evaluation of therapeutic efficacy in CCl4 and paracetamol-induced acute liver failure models via mesenchymal stem cell-derived hepatic spheroid delivery.	hyaluronic acid/gelatin/sodium alginate scaffold with N-acetylcysteine (NAC)-capped gold nanoclusters	Composite significantly reduced serum ALT/AST levels and improved liver histopathology, effectively treating acute liver failure in mouse models.
[64]	Pigs; SD rats	Endoscopic gastric ulcer sealing in pigs and assessment of MRSA-infected full-thickness skin wound healing in a rat model.	Acryl aspartate (AASP) and cysteine-grafted carboxymethyl chitosan (CMCS-NAC) as the base matrix, integrated with gastric acid-responsive charge-reversal antibacterial molecules	Achieved immediate and sustained gastric mucosal adhesion and effectively eradicated MRSA, significantly accelerating the healing of infected wounds.
[84]	C57BL/6 mice	Biocompatibility assessment through dorsal subcutaneous implantation, focusing on inflammatory response and local tissue integration over a four-week period.	Methacrylated hyaluronic acid crosslinked with NAC	Showed excellent biocompatibility with mild initial inflammation that resolved completely by week four, demonstrating no adverse effect on organisms.
[17]	SD rats	Hemostatic performance evaluation using liver incision and femoral artery transection models to measure clotting time and total blood loss.	GelMA-Gel/NAC	GelMA-Gel/NAC significantly shortened hemostasis time and reduced blood loss compared to commercial gelatin sponges in both rat models.
[44]	SD rats (diabetic)	STZ-induced diabetic rat model with full-thickness wounds to evaluate basic fibroblast growth factor release on re-epithelialization and angiogenesis.	Thiolated Chitosan-dextran, using NAC	Accelerated wound contraction and promoted granulation tissue formation and angiogenesis through up-regulation of PCNA and VEGF expression.
[52]	SD rats	Investigation of brain-targeted drug delivery through nasal cavity administration, assessing hydrogel retention and potential local tissue toxicity.	Hyaluronic acid/silk fibroin/dopamine/NAC hydrogel	Successfully delivered NAC to brain regions via the nasal cavity without inducing damage to olfactory bulbs or hippocampus tissues.
[87]	C57BL/6 mice (diabetic)	Evaluation of diabetic wound healing efficiency using a full-thickness skin defect model with sequential release of NAC and bFGF.	bFGF-HSPP-NAC	Sequential release effectively mitigated inflammation and promoted rapid re-epithelialization, leading to complete wound healing by day seventeen.
[56]	C57BL/6J mice (diabetic)	Assessment of long-term blood glucose regulation following transplantation of islet-laden microgels into the epididymal fat pad of diabetic mice.	Combination of Maleimide grafted and thiol (NAC) grafted chitosan	Maintained stable blood glucose levels for over 120 days while protecting transplanted islets from host immune-mediated foreign body reactions.
[78]	C57BL/6J mice	Middle ear injection to evaluate prevention of cisplatin-induced hearing loss by maintaining mitochondrial homeostasis and hair cell survival.	GelMA containing encapsulated NAC	Effectively prevented cisplatin-induced hearing loss and hair cell damage by activating the PI3K/AKT/mTOR pathway and remodeling mitochondrial homeostasis.
[63]	KM mice	Protective effects against alcohol-induced acute and chronic gastric mucosal injury evaluated via gavage and histopathological scoring of lesions.	CS-NAC/ALG containing tilapia collagen peptide	Reduced gastric injury area and inflammatory markers while significantly improving antioxidant enzyme activities (SOD, CAT, GSH) in the stomach.
[73]	SD rats	Functional recovery assessment in a 10 mm sciatic nerve gap model using conduits with physical, chemical, and therapeutic cues.	Gellan–Xanthan conduit	Promoted significant functional nerve regeneration and re-innervation, as evidenced by improved walking track analysis and gastrocnemius muscle mass.
[15]	BALB/c mice	Full-thickness skin wound model comparing re-epithelialization rates and collagen deposition quality between experimental hydrogels and commercial dressings.	Methacrylated gelatin–NAC	Accelerated wound closure by 3.37 times compared to untreated groups and promoted regenerative healing with organized collagen and diminished fibrosis.
[83]	Mice	Subdermal implantation study to monitor in vivo degradation rates and local tissue inflammatory responses over a 28-day study period.	Thiolated Chitosan–NAC combined with oxidized dextran	Demonstrated high biocompatibility with no signs of edema or necrosis and exhibited gradual, controlled degradation without adverse systemic effects.
[10]	BALB/c and ICR mice	Evaluation of synergistic chemo-photodynamic therapy in breast cancer models and anti-inflammatory pro-healing effects in second-degree burn wounds.	Modified Hyaluronic acid, amine crosslinked containing NAC	Achieved enhanced anti-tumor efficacy in 4T1 models and promoted rapid collagen deposition and tissue regeneration in burn wound healing.
[30]	SD rats	Assessment of scaffold degradability via subcutaneous pockets and cartilage regeneration in distal femoral condyle defects over four weeks.	Thiol chitosan–Poly(lactic-co-glycolic acid)–Polydopamine	Effectively bridged cartilage defects with newly formed tissue, resulting in significantly higher histological regeneration scores compared to control groups.
[25]	Diabetic mice	Infected diabetic wound model to test synergistic antibacterial and antioxidant functions under near-infrared irradiation to promote healing.	Molybdenum-functionalized chitosan, crosslinked with a PEG derivative	Synergistic photothermal and antioxidant therapy successfully eradicated bacteria and promoted rapid healing of infected diabetic skin wounds.
[65]	NZW rabbits	Ocular safety and tolerability assessment through instillation into the inferior fornix, evaluating corneal health and anterior chamber inflammation.	poly(n-isopropylacrylamide) crosslinked with chitosan, NAC	Proved safe and well-tolerated, inducing no corneal damage or inflammatory cell infiltration after four days of continuous ocular exposure.
[96]	ICR mice (diabetic)	STZ-induced diabetic model with full-thickness wounds to test multifunctional dressings with on-demand degradation and antioxidant properties.	Modified PEG/chitosan crosslinked, NAC	Significantly enhanced the wound healing rate in diabetic mice through synergistic antioxidant and antimicrobial effects compared to saline controls.
[32]	SD rats	Hemostatic evaluation via tail amputation and infected wound healing assay involving macrophage polarization and self-supplied H2S release.	Oxidized hyaluronic acid/NAC combined with modified chitosan	Accelerated hemostasis and promoted infected wound healing by enhancing M2 macrophage polarization and increasing CD31-positive blood vessel density.
[36]	SD rats	Evaluation of scarless healing in 20 mm full-thickness skin incisions, focusing on collagen organization and wound closure speed.	collagen–GO-loaded NAC	Promoted rapid wound closure and scarless skin regeneration by improving collagen alignment and enhancing the local microenvironment during healing.
[61]	Kunming mice	Protection against alcohol-induced acute liver injury and chronic brain injury assessed through serum biochemistry and Morris water maze.	Sulfhydryl functionalized chitosan/alginate/peptide	Mitigated elevation of liver enzymes (ALT/AST) and significantly improved spatial memory and learning ability in alcohol-injured mice.
[66]	Wistar rats (diabetic)	Intravitreal injection model for diabetic retinopathy to evaluate anti-angiogenic effects and down-regulation of retinal VEGF expression levels.	Avastin/dexamethasone/chitosan–NAC	Successfully inhibited retinal neovascularization and hemorrhage while significantly down-regulating VEGF expression in diabetic retinopathy rat models.
[67]	Rabbits	Established implant-related *S. aureus* tibia infection model used to evaluate local vancomycin release for infection prophylaxis.	Vancomycin-loaded gel	Proved highly effective for prophylaxis, with vancomycin-loaded groups remaining culture-negative and showing significantly lower infection and inflammation scores.
[33]	SD rats	Evaluation of therapeutic effects in an oval full-thickness skin excision model, monitoring wound closure and histological regeneration.	PCL-Col/NAC scaffold	Provided sustained NAC release, achieving 93.59% wound closure by day twelve and significantly increasing the thickness of the epidermal layer.

## Data Availability

No new data were created or analyzed in this study. Data sharing is not applicable to this article.

## Supplementary Materials

The following supporting information can be downloaded at: https://www.mdpi.com/article/10.3390/gels12080751/s1, PRISMA_2020_Checklist [102].

## Abbreviations

The following abbreviations are used in this manuscript:

(NacMDP)(Ch-SS)	Chitosan modified with N-acetyl-L-cysteine 3-((2acetamido-3-methoxy-3-oxopropyl)dithio) propanoic acid
(PE(NAC)4)	Pentaerythritol-based tetrathiol crosslinker (PE(NAC)4)
(PEA) diol	Poly (ethylene adipate) diol
•OH	Hydroxydyl radicals
4-HNE	4-hydroxynonenal
5-FU	5-fluorouracil
8-OHdG	8-hydroxy-2′-deoxyguanosine
AA	Acrylic acid
Aam	Oracrylamide
AA-PANi-Q	Asiatic acid-poly(aniline)-quercetin
AASP	N-acryloyl aspartame
ABTS assay	2,2′-azino-bis(3-ethylbenzothiazoline-6-sulfonic acid)
AC16	Human CMs
AgNW	Silver nanowire
AI	Artificial Intelligence
ALA	5-aminolevulinic
Alg	Alginate
Alg-SH	Thiol-functionalized alginate
ALT	Alanine aminotransferase
AML12	Alpha Mouse Liver 12 cells
APG	Polyethylene glycol modified with 4-formylbenzoic acid
APS	Ammonium persulfate
AS-HA	Aldehyde-modified hyaluronic acid
AST	Aspartate aminotransferase
ATP	Adenosine triphosphate
BALB/c	Bagg Albino mouse line cells
BDDE	1,4-butane-diol diglycidyl ether
bEnd.3	Mouse brain microvascular endothelial line cells
Bfgf	Basic fibroblast growth factor
BHI	Brain heart infusion
BJ1	Human normal fibroblast cell line
BMSCs	Bone marrow mesenchymal stem cells
BSA	Bovine serum albumin
C_16_N-DCA	Charge-reversal antibacterial molecules
C57BL/6	C57 black 6 (inbred mouse strain)
CAT	Enzyme catalase
CCD-1112SK	Human skin fibroblasts cells
CCl4	Carbon tetrachloride
CD31	Cluster of Differentiation 31 protein
CEX	Celecoxib
CFU	Colony-forming unit
ChMA	Chitosan methacrylate
CL-LA-PEG200	Ε-Caprolactone, Rac-Lactide and Poly(ethylene glycol) copolymer
CL-LA-PEG200-PEA	Copolymer of Caprolactone, lactic-acid, poly(ethilene glycol)-poly(ethylene adipate)
CMC	Carboxymethyl cellulose
CMCS	Carboxymethyl chitosan
CMC-SH	Thiol-modified carboxymethyl cellulose
CMCS-NAC	NAC-modified carboxymethyl chitosan
CMHA-S	Thiol-modified carboxymethyl hyaluronic acid
Col	Collagen
-COOH	Carboxylic acid group
CRL-2522	Human fibroblast cells
CS	Chitosan
CS-Cys	Chitosan modified with cysteine
CSDS	Catechol-conjugated chitosan modified with thiol group
CS-Mal	Maleimide-grafted chitosan
CS-NAC	Chitosan modified with NAC
CS-NAC-β-lg	Chitosan modified with NAC and β-lactoglobulin
CS-SH	Thiol-modified chitosan
CS-SNO	Chitosan modified with NAC and NO group
CTS-pNAMP-NAC3	(Chitosan-poly(n-isopropylacrylamide-co-acrylic acid-co-methyl methacrylate-co-pyridyl disulfide ethylmethacrylate)-acetylcysteine)
Cys	Cystamine dihydrochloride
DA	Dopamine
DAC	Disposable Antibacterial Coating
Dex-Ma	Maleic acid modified dextran
DPPH	2,2-diphenyl-1-picrylhydrazyl ou 2,2-diphenyl-1-picrylhydrazyl-hydrate
DS	Degree of substitution
EDC	1-ethyl-3-(3-dimethylaminopropyl)-carbodiimide hydrochloride
EGCG	(-)-Epi-gallocatechin gallate
EG-Cu-CA NPs	(-)-epi-gallocatechin gallate (EGCG) -copper ionic-κ-carrageenan (κ-CA) nanoparticles
EGDMA	Ethylene glycol dimethacrylate
ELISA	Enzyme-Linked Immunosorbent Assay
EPLM	Maleimide group modified ε-polylysine
EPL-PEG-MAL	Polyethylene glycol-maleimide-modified polypeptide
EWPD	Egg white-derived peptides
FCS	Fetal calf serum
FITC	Fluorescein isothiocyanate isomer
FTIR	Fourier transform infrared spectroscopy
GA	Glutaraldehyde
GC-NAC-MNA	NAC-glycol-chitosan
Gel	Gelatin
GelMA	Gelatin Methacryloyl
Gel-NAC	NAC-modified gelatin (Gel-SH)
Gel-Ty	Gelatin modified with tyramine
GEM	Gemcitabine
GG	Guar gum
G-NAC	NAC grafted on gold nanoparticles
GNP	Gold nanoparticles
GO	Graphene oxide
GP	Genipin
GSH	Glutathione
Gtn-DTPH	Thiol-modified gelatin (Gtn-DTPH)
H	2-hydroxyethyl methacrylate (Gel-HP-NAC)
H_2_S	Hydrogen sulfide
HA	Hyaluronic acid
HaCat	Human keratinocytes cells
HAMA-Cat	Hyaluronic acid methacrylated modified with dopamine
HA-NH2	Hyaluronic acid modified with ethane diamine
hASCs	Human adipose-derived stem cells
HA-SH	Cysteine-modified sodium hyaluronato
hBMSCs	Human buccal pouch stem cells
HCA2	Dermal fibroblasts
HCECs	Human corneal epithelial cells
HDFs	Human dermal fibroblasts
HDI	1,6-diisocyanatohexane (hexamethylene diisocyanate
HEI-OC1	House Ear Institute-Organ of Corti mouse cells
HEMA	2-hydroxyethyl methacrylate
HepG2	Human hepatocarcinoma cells
HGF-1	Human gingival fibroblasts
HO-1	Heme oxygenase-1
HPLC	High Performance Liquid Chromatography
HPMC	Hydroxypropyl methylcellulose
Hp-β-CD/CEX	Inclusion complex 2-Hydroxypropyl-β-cyclodextrin/Celecoxib (Hp-β-CD/CEX)
HRP	Horseradish peroxidase
HS	Human serum
HUVECs	Umbilical vein endothelial
IC_50_	Half-maximal inhibitory concentration
ICP-AES	Inductively coupled plasma atomic emission spectroscopy
ICR mice	Institute of Cancer Research outbred mouse
IFP-MSCs	Infrapatellar fat pad-derived mesenchymal stem cells
IL-6	Interleucina-6
IOSE80 cells	Epithelial cells
IRG2959	2-hydroxy-4′-(2-hydroxyethoxy)-2-methylpropiophenone
KCP	*Klebsiella pneumoniae*
LO2	Human hepatic cell line
L929	L929 fibroblast
LAP	Lithium phenyl-2,4,6-trimethylbenzoylphosphinate
Lys	Lysine
MBA	N.N-methylenebisacrylamide
MBG/SHP	Strontium-substituted mesoporous bioactive glass
MBG_Sr_NAC	NAC-loaded strontium-substituted mesoporous bioactive glass
MC	methylcellulose
MC3T3 cells	Osteoblasts
Mcol	Marine collagen
MgOL	Magnesium oleate
MIC	Minimum inhibitory concentration
MMP2	Matrix metalloproteinase-2
MMP9	Matrix metalloproteinase-9
MoS_2_NS	Molybdenum disulfide nanosheets
MRSA	Methicillin-resistant *Staphylococcus aureus*
N. R.	Not informed
N.P.	Not performed
NAC	N-acetylcysteine
NAC CPDs	N-acetyl-l-cysteine-derived carbonized polymer dots
NAC-Au NCs	NAC-capped gold nanoclusters
NAC-CS-PCL	NAC modified chitosan-co-polycaprolactone
NaCl	Sodium chloride
NaHA	Sodium hyaluronate
NaHA-NAC	Hyaluronic acid sodium salt modified by NAC
NAPT	Nucleic Acid Purification and Testing
NCI-H460	Human lung cancer cell line
NGF	Nerve growth factor
NHS	N-hydroxy succinimide system
NIH 3 T3	Mouse embryonic fibroblasts
NIPA	N-isopropylacrylamide
NIR	Near-infrared irradiation
NMR	Nuclear Magnetic Resonance
NQO1	NAD(P)H Quinone Dehydrogenase 1
NRF2	Nuclear factor erythroid 2-related factor 2
NZW rabbits	New Zealand White rabbits
O_2_•^−^	Superoxide anions
Odex	Partially oxidized dextran
OHA	Oxidized hyaluronic acid
P	Poly(ethylene glycol) methyl ether methacrylate (Gel-HP-NAC)
PA	Polyamide nanofibers (PAs)
PAMP	Pro-angiogenic peptide derived from adrenomedullin
PBS	Phosphate-buffered solution
PCL	Poly (ε-caprolactone)
PCNA	Proliferating Cell Nuclear Antigen
PD	Polydopamine
PDA-PEG	Pyridyl disulfide acrylate (PDA) and poly(ethylene glycol) methyl ether methacrylate (PEGMA) copolymer
PEG	Polyethylene glycol
PEG-4mal	Poly(ethylene glycol) tetra maleimide
PEG-4SH	Poly(ethylene glycol) tetra thiol
PEGDA	Polyethylene glycol diacrylate
PEGDT	Poly(ethylene glycol) dithiol
PEGMA	Poly(ethylene glycol) methyl ether methacrylate
PF127	Poloxamer
PHBV	Poly(3-hydroxybutyric acid-co3-hydroxyvaleric acid)
PI3K/AKT/mTOR	Phosphoinositide 3-kinase/protein kinase B/mechanistic target of rapamycin signaling pathway
PIM(Cn)-Mal	Polyimidazolium-containing maleimide terminal groups
PLA	Polylatic acid
PLGA	Poly (lactic-co-glycolic acid)
PLGA-PDA	Dopamine-modified Poly (lactic-co-glycolic acid)
PMMA	Polymethylmethacrylate
PMMA	Poly(methyl methacrylate)
POSS-PEG-CHO	Benzaldehyde-terminated polyethylene glycol
PU	Polyurethane
PVA	Poly(vinyl alcohol)
PVB	Poly(vinyl butyral)
rASCs	Rat adipose stem cells
ROS	Reactive oxygen species
RP-HPLC	Reverse-phase high-performance liquid chromatography
SA	Sodium alginate
SA-HA	Aldehyde-modified hyaluronic acid
SBF	Simulated body fluid
SD rats	Sprague-Dawley rats
SDS-PAGE	Sodium dodecyl sulfate polyacrylamide gel electrophoresis
SF	Methacrylate silk fibroin
SGF	Simulated gastric fluid
-SH	Sulfhydryl group
SHP	Poly(ether urethane)-based hydrogel
SIF	Simulated intestinal fluid
SOD	Antioxidant enzymes superoxide dismutase
Sr^2+^	Strontium ion
SWF	Simulated wound fluid
TA	Tannic acid
TCP	Tilapia collagen peptide
THP-1 cells	Human acute monocytic leukemia cell line
TK-NH2	Thioketal linker with amino end groups
TNC	NAC-modified poly(N-isopropylacrylamide)-g-chitosan
TNF-α	Tumor necrosis factor-alpha
TNF-β	Tumor necrosis factor-beta
UV-Vis	Ultraviolet-visible
VA-086	2,2′-azobis[2-methyl-n-(2-hydroxyethyl)propionamide]
VEGF	Vascular endothelial growth factor
XPS	X-Ray Photoelectron Spectroscopy
Zr-MOF-Mn	Zr-organic frame-Mn
β-GP	β-glycerol phosphate
β-lg	β-lactoblobulin
γH2AX	Phosphorylated histone H2AX
ε-PL	ε-Poly-L-lysine
ε-PL-SATO	ε-poly-L-lysine-S-Aroylthiooximes

## Appendix A

**Table A1:** Bioactivity of hydrogels containing NAC. N.I.—Not informed; N.A—Not applied.

Ref	Components	Degree of Thiolation	Gelation	Role of NAC	Microbiological and Biocompatibility Models	Effects	Application Intended
[74]	NAC–glycol chitosan (GC-NAC-MNA), Pluronic F127, Gemcitabine (GEM) and inclusion complex 2-Hydroxypropyl-β-cyclodextrin/Celecoxib (Hp-β-CD/CEX)	6.3 mmol/g	Chemical, 37 °C, 30 s	Modifying agent	Freshly excised pig bladders.	Temperature-responsive, good adhesion to bladder tissue, sustained-drug delivery.	Bladder cancer treatment
[72]	CS-NAC or chitosan modified with N-acetyl-L-cysteine 3-((2acetamido-3-methoxy-3-oxopropyl)dithio) propanoic acid (NacMDP) (Ch-SS), heparin	1.9–2.7 μmol/g	Chemical	Modifying agent	3T3 cells.	Biocompatibility, cell adhesion, and proliferation.	Brain injury treatment and peripheral nerve regeneration
[73]	Gellan gum, xanthan gum, propylene glycol, CaCl_2_, DS, PMMA, nerve growth factor (NGF), NAC, Magnesium oleate (MgOL), poly(3-hydroxybutyric acid-co3-hydroxyvaleric acid) (PHBV)	N.A.	Chemical	Modifying agent	The rat adrenal gland pheochromocytoma PC12 (CPC-12C); Male and female SD rats	Bioactivity, biocompatibility, nerve repair and reduction in muscle mass atrophy Fibers containing NAC improved neurite extension compared to no-NAC fibers.	
[75]	CS-NAC, norbornene functionalized chitosan (NorCS), SA, L2959, CaCl_2_	17%	UV, 365 nm, 3 s	Modifying agent, crosslinked.	hBMSCs; Functional assessment of cartilage replacement at bilateral knee joints in mice	Biocompatibility, biodegradability, self-healing, injectability, and cartilage reconstruction.	Cartilage regeneration
[76]	Polyurethane (PU), NAC, Alg, Col, GP	47–80%	Chemical, 6 h	Modifying agent	Rat adipose stem cells (rASCs), chondrogenesis differentiation: static and dynamic compression culture.	Biocompatible, high mechanical resistance, and in vitro expression of SOX-9, Aggrecan in dynamic compression culture.	
[51]	Hyaluronic acid sodium salt modified by NAC (NaHA-NAC)	N.A.	Chemical, 10–6.5 h depending on the pH	Modifying agent, crosslinked	N.A.	pH-dependent gelation	Drug delivery system and cell incubation
[53]	Poly(ethylene glycol) dithiol (PEGDT), Eosin Y or 2-hydroxy-4′-(2-hydroxyethoxy)-2-methylpropiophenone (IRG2959), NAC Polymers synthesized by the authors: poly(dimethylamino ethyl methacrylate) (P1 or PDMAEMA); poly(2-butenyl-2-oxazoline) (P2); poly(methyl vinyl ether alt-maleic anhydride((P3); Poly(acrylic acid-co-pentenyl acrylate) (P4); carboxymetyl cellulose allyl ester (P5); norbornene-carboxymethyl cellulose (P6); norbornene-functionalized hyaluronic acid (P7)	N.A.	UV-vis, 120–150 s	Crosslinker	HUVECs, dermal fibroblasts (HCA2) (in vitro).	Low cytotoxicity. Polymers with strong electrostatic charge density were more toxic to cells. pH-dependent coupling, most reactions between NAC and polymers containing terminal C=C are more efficient at lower pH.	
[55]	Gel, alginate (Alg), NAC, Tiron, AC16 human CMs	N.I.	Physical (CaCl_2_), 15 min	Loaded into the hydrogel	AC16 human CMs.	NAC’s ROS scavenging effect mitigated the oxidative stress induced by doxorubicin (DOX).	
[54]	CS-NAC or chitosan modified with cysteine (CS-Cys), β-lactoglobulin (β-lg), glutaraldehyde (GA), egg white-derived peptides (EWDP), curcumin	N.I.	Chemical, 10 min	Modifying agent	N.A.	The drug release was controlled and stained at a lower pH.	
[58]	Insulin, CS-NAC, Bovine serum albumin (BSA)	210.6–321.4 μmol/g	Chemical, 37 °C	Modifying agent, crosslinked.	NIH 3T3 cells.	Biocompatibility, sustained protein release. Cytotoxicity increased with an increase in the amount of thiol present on the chitosan chains. The cells migrated and adhered to the hydrogel networks.	
[52]	Dopamine (DA), Hyaluronic acid (HA), NAC, silk fibroin, 1,4-butanediol diglycidyl ether (BDDE) H_2_O_2_	N.A.	Chemical, 37 °C, 1–1.5 h	Loaded into the hydrogel	L929 cells; Male SD rats.	In vitro, NAC delivery increased when NIR irradiation was applied. In vivo, NAC was delivered to the brain tissue and retained in the nasal cavity for 120 min, and its effect was enhanced by NIR irradiation.	
[56]	Maleimide grafted chitosan (CS-Mal), CS-NAC, thiol-modified carboxymethyl cellulose (CMC-SH), BSA, and islet cells	261 μmol/g (CS-NAC), 664 μmol/g (CMC-SH)	Chemical room temperature (r.t.), 60 s	Modifying agent, crosslinked.	8 weeks old male SD rats (for islets isolation); 1–8 weeks old C57BL/6J male mice (T1DM in vivo treatment).	Good cell compatibility, excellent hemocompatibility, good glycemic regulation. The chitosan-derivative microgels were stable for 7 days in PBS and lysozyme solutions. CMC-SH scaffolds containing CS-NAC and CS-Mal microgels reduced the expression of TNF-α, iNOS, IL-1β, and IL-6, which are found in the inflammatory response. The encapsulated islet cells showed viability for 7 days.	
[94]	Catechol-chitosan (CCS), CS-NAC, FeCl_3_, NaIO_4_, doxorubicin (DOX)	24.5%	Chemical, rt., 45–1874 s	Modifying agent	The human lung cancer cell line NCI-H460.	Sustained drug release, fast gelation, and biocompatibility.	
[57]	NAC-modified poly(N-isopropylacrylamide)-g-chitosan (TNC)	299.39 ± 8.11 μmol/g	Chemical, 37 °C, 10 min	Modifying agent, crosslinked	Infrapatellar fat pad-derived mesenchymal stem cells (IFP-MSCs), NIH-3T3, and osteoblasts (MC3T3-E1).	Non-cytotoxic, fast gelation, body-temperature gelation, injectable, thermosensitive All three cell types displayed progressive growth over 7 days.	
[77]	NAC, CS, borax, Polyvinyl alcohol (PVA)	N.A.	Chemical, <1 min	Loaded into the hydrogel	N.I.	Sustained drug delivery	Hearing loss prevention
[78]	GelMA, LAP, -acetyl-l-cysteine-derived carbonized polymer dots (NAC CPDs), and manganese porphyrin	N.A.	UV, 405 nm, 10 s	Loaded onto the nanoparticles	HEI-OC1 mouse cochlear HCs; 6–8 weeks old C57BL/6J wild-type male and female mice.	Injectability, high adhesiveness, sustained release, and antioxidant and anti-inflammatory properties.	
[59]	NAC-capped gold nanoclusters (NAC-Au NCs), NaHA, Gel, SA	N.A.	Physical	Loaded onto nanoparticles	AML12 and LO2 cells; 6–8 weeks old male C57BL/6 mice.	Antioxidant and anti-inflammatory activities, angiogenesis, and high cell viability. NAC-capped gold nanoclusters, by themselves, displayed cell viability after 12 h, from 50 μg/mL to 500 μg/mL. The hydrogel containing NAC-Au NCs showed a decrease in ROS levels in the liver tissue. In vivo, the composite showed evidence of microvessel formation after three days of implantation.	Liver injury Treatment
[61]	CS-NAC, nano-CaCO_3_, TCP, SA	9%	Physical	Modifying agent	28–35 weeks old ale Kunming mice. Liver injury: (10 mL per kg per BW) once a day for 7 days. Brain injury: 56 KingDrink (2 mL per kg per BW) for 8 weeks.	Antioxidant activity, reducing alcoholic liver and brain injury. The activities of ADH and ALDH, alcohol metabolic enzymes, increased significantly when hydrogels were applied in vivo. The hydrogel groups, with and without TCP, displayed an improvement in memory acquisition function in alcohol-injured mice.	
[60]	Commercial bioink based on gelatin methacrylate (GelMA), Alg, xanthan gum and LAMININ a5b2y1 (GelXA LAMININK), CaCl_2_, NAC, Human hepatocarcinoma cells (HepG2 cells)	N.A.	Physical, 5 min	Loaded into the hydrogel	HepG2 cells (encapsulated in the scaffolds).	Anti-inflammatory activity and reduction in hepatotoxicity. The 3D bioprinted hepatic model treated with NAC was able to protect against paracetamol toxicity in vitro and made the spinning cell cultures more susceptible and responsive to drugs than static cultures.	
[66]	Chitosan-N-acetyl-L-cysteine nanoparticles containing Bevacizumab (Avastin), Poloxamer (PF127), and hydroxypropyl methylcellulose (HPMC), Sodium tripolyphosphate (TPP)	32%	Physical, 37 °C, 165 s	Modifying agent	HUVECs; Adult male Wistar rats, blood glucose levels of 250 mg/dL.	Injectability, slow drug release, drug encapsulation, and high cell viability.	Ocular treatment
[65]	Chitosan-Poly(n-isopropylacrylamide-co-acrylic acid co-methyl methacrylate-co-pyridyl disulfide ethyl methacrylate) (CTS-pNAMP), NAC, atropine	N.I.	Physical, r. t., 24 h	Crosslinker	Human corneal epithelial cells (HCECs); 35 weeks old female New Zealand White rabbits.	Cell viability was similar to that of the control group after 24 h in vitro and after 4 days in vivo. Good biocompatibility in vitro and in vivo was observed.	
[69]	Gold Nano Particles (GNP) or NAC grafted on gold nano particles (G-NAC), Gelatin modified with tyramine (Gel-Ty), Horseradish peroxidase (HRP), and hydrogen peroxide (H_2_O_2_)	N.I.	Chemical, 1 min	Loaded onto the nano- particles	Human adipose-derived stem cells (hASCs).	Cell viability and osteodifferentiation.	Osteoregeneration and bone defect treatment
[70]	NAC-loaded strontium-substituted mesoporous bioactive glass (MBG_Sr_NAC), Poly(ether urethane)-based hydrogel (SHP) GelMA, chitosan methacrylate (ChMA), LAP, NAC and/or pro-angiogenic peptide derived from adrenomedullin (PAMP)	N.A. N.A.	Physical, 37 °C, 15 min UV, 60 s	Loaded onto the nanoparticles Loaded into the hydrogel	L929 and osteoblast-like SAOS2 cells Pre-osteoblastic MC3T3-E1 cells, bone marrow from an 8 weeks old mouse femur and tibia (in situ); Calvaria bone from a four-day-old mouse (ex vivo)	NAC sustained release, thermosensitive gelation. Strontium-substituted mesoporous bioactive glass exhibits bioactivity properties in vitro. However, hydrogels containing NAC-loaded nanoparticles have not been tested. High biocompatibility, bone mineralization ex vivo, potential osteo differentiation, sustained drug delivery.	
[67]	Commercial hydrogel based on HA and polylactic acid (PLA)	N.A.	N.I.	Loaded into the hydrogel	16 weeks old female New Zeeland White rabbits with a sand-blasted titanium rod (diameter 4 mm, length 25 mm), implanted in the medullary canal of the left tibia, infected with *S. aureus*.	NA-loaded hydrogels applied to implants showed results similar to those of the no-drug-loaded hydrogel. The concentration used (0.5% *w*/*v* NAC) was previously effective in vitro against *S. epidermidis* and *S. aureus*. The authors considered that the NAC concentration might have been insufficient for the treatment.	
[71]	Commercial collagen membrane (Tissue Guide) and spongy scaffold made from bovine type I atelocollagen (Collaplug), NAC.	N.A.	N.I.	Loaded into the hydrogel	*S. aureus* 209P and *S. pyogenes*. Fibroblastic and bone marrow cells from the palatal gingiva of 8 weeks old SD rats.	Anti-inflammatory activity, bacteriostatic activity, oxidative stress reduction, and biocompatibility. Cell viability was evaluated in bacteria and a cell co-culture model. Preloading the hydrogels with NAC prevented the effects of bacterial infection on cell viability, fibroblastic attachment, adhesion, and proliferation.	
[103]	NAC, Bovine conditioned medium type I (Cytoplast^®^ RTM), human DFDB (DynaGraft-D™)	N.I.	N.I.	Loaded into the hydrogel	Calvarial osteoblasts from the parietal or the frontal bones of 8 weeks old SD male rats.	Biocompatibility, high cell adhesion, antioxidant and anti-inflammatory activity	
[68]	Commercial Disposable Antibacterial Coating (DAC) hydrogel, NAC	N.I.	N.I.	Loaded into the hydrogel	*S. aureus*, *S. epidermidis*, *E. coli*, *E. faecalis*, *A. baumannii*, and *P. aeruginosa*; New Zealand White rabbits’ tibia.	Rapid reabsorption, drug release, shear resistance for chirurgical implantation, bactericidal activity, antibiofilm activity. NAC’s minimum inhibitory concentration (MIC) is lower in hydrogel systems (6.125 mg/mL) than in NAC alone.	
[104]	N-isopropylacrylamide (NIPA) oracrylamide (AAm),N.N-methylenebisacrylamide (MBA) and 2,2-dieth DEAP, NAC, and bovine hemoglobin	N.I.	UV	Loaded into the hydrogel	N.A.	Oxygen transportation. NAC was used as a reducing agent that was co-encapsulated with hemoglobin.	Oxygen carrier
[79]	GelMA, 2,2′-azobis[2-methyl-n-(2-hydroxyethyl)propionamide] (VA-086), MG63 osteosarcoma cell	N.A.	UV, 440 nm, 1–4 min	Loaded into the hydrogel	MG63 osteosarcoma cell	Antioxidant activity, high cell proliferation, and cell viability. Pre-treatment with 10 mM NAC reduced ROS levels after 2 and 4 min of irradiation.	Periodontal treatment
[84]	HAMA, pentaerythritol-based tetrathiol crosslinker (PE(NAC)_4_)	N.A.	UV, 405 nm, 15–150 s	Crosslinker	HUVECs; C57BL/6 mice	Good biodegradability, biocompatibility, cell growth, precision patterning, and vascular endothelial growth factor (VEGF) release.	Tissue engineering
[85]	CS-NAC and e-polylysine polyethylene glycol-maleimide (EPL-PEG-MAL)	159.4 μmol/g	Chemical, 1 min	Modifying agent, crosslinked	L929 cells.	Non-cytotoxicity, good water uptake, high storage modulus, adhesiveness, and rapid gelation.	
[81]	CS-NAC and Poly(ethylene glycol) diacrylate (PEGDA)	221.0–361.4 umol/g	Chemical, 37 °C, 25–130 min	Modifying agent, crosslinked	HDFs and A549 cells.	Non-cytotoxicity in vitro, good water absorption, good porosity, short time gelation, and degradable in physiological conditions.	
[80]	CMHA-S and Gtn-DTPH coating Sephadex G-50 beads	N.I.	Chemical, overnight	Loaded into the hydrogel	Int-407 cells	Cell growth and cytocompatibility. NAC treatment triggered hydrogel dissolution.	
[82]	HAMA, hyaluronic acid methacrylated modified with dopamine (HAMA-Cat), LAP and PE(NAC)_4_)	N.A.	UV, 405 nm, 18–32 s	Crosslinker	NH3T3, L929 cells and HUVECs	Good adhesion, low cytotoxicity, good compressive strength, promotes cell growth, adhesion and differentiation, and printability.	
[90]	CS-NAC, PEGDMA, I2959	312.6 μmol/g	UV, 30–530 s	Modifying agent	L929 cells	Non-cytotoxicity.	
[86]	NAC, silver salts	N.A.	Physical, r. t., 3 h	Complexation agent	Wi-38 human normal embryonic lung cells; *S. aureus*, *A. baumannii*, *P. aeruginosa* and *S. intermedius*.	Antibacterial and antibiofilm activities.	
[17]	GelMA, Gel, 2-hydroxy-2-methylphenylacetone (I2959), NAC	N.A.	UV, 362 nm, 40 min	Loaded into the hydrogel	6 weeks old male SD rats	Good in vitro coagulation, good adhesion, and blood compatibility.	
[83]	CS-NAC, partially oxidized dextran (Odex)	8.1–40.8%	Chemical, 37 °C, 10 s	Modifying agent	Dermal fibroblasts; mice.	Biocompatibility, good in vivo durability.	
[64]	NAC-modified carboxymethyl chitosan (CMCS-NAC), N-acryloyl aspartate (AASP), charge-reversal antibacterial molecules (C_16_N-DCA), LAP		UV, 5–20 s	Modifying agent	*E. coli*, *S. aureus*, *H. Pylori*.	Self-healing, anti-swelling performance, strong adhesion within 5 s, lasting 24 h, adhesion in pig skin and stomach after 24 h of immersion in PBS, minimizes ulcer contact with the stomach acid and promotes speedy healing, and antibacterial activity attributed to the C_16_N-DCA molecule and CMCS chain.	Ulcer treatment
[62]	Carboxymethyl cellulose (CMC), Gel, Alg, NAC, CaCl_2_	N.A.	Physical	Loaded into the hydrogel	Anticoagulated human blood; NIH3T3 cells; Adult Wistar male rats, pressure ulcer induced.	Sustained drug release, antioxidant activity, anti-inflammatory activity, cytocompatibility, and blood compatibility. Histopathological analysis showed re-epithelialization and regeneration of the dermis, epidermis, sebaceous glands, and hair follicles in the injuries treated with the NAC-containing hydrogel.	
[63]	CS-NAC, nano CaCO_3_, Alg, Tilapia collagen peptide	9%	Physical	Modifying agent	4–5 weeks old KM male mice, Red Star Liquor (56% alcohol *v*/*v*), 13 mL/kg-BW to induce gastric mucosal injury.	pH-responsive gelation, antioxidant activity, anti-inflammatory activity, biodegradability, and strong mucoadhesion. The hydrogel displayed a significant increase in the levels of protective enzymes (SOD, GSH, and CAT). An increase in the activity of liver enzymes (alcohol dehydrogenase and aldehyde dehydrogenase) was also observed	
[16]	NAC, methylcellulose (MC)	N.A.	Physical, 37 °C, 5 min	Loaded into the hydrogel	Human fibroblast cells (CRL-2522); 6 weeks old SD male rats, ulcer chemically induced by 60% acetic acid topical application	Thermo-responsive gelation, cell viability, dose-dependent, ulcer size reduction, anti-inflammation activity, and antioxidant effect.	
[15]	Porcine skin gelatin, 2-hydroxyethyl methacrylate (HEMA), poly(ethylene glycol) methyl ether methacrylate (PEGMA), NAC, ethylene glycol dimethacrylate (EGDMA) crosslinker, and ammonium persulfate (APS) thermal initiator.	N.A.	Chemical, 22 °C	Loaded in the hydrogel, it may act as a crosslinker	Human skin fibroblasts cells, CCD-1112SK	Hemocompatibility, ability to promote blood coagulation (hemostasis), anti-inflammatory properties, and induction of accelerated re-epithelialization.	Wound healing
[42]	Chitosan (CS), collagen (Col), NAC, ε-Poly-L-lysine (ε-PL)	N.I.	Chemical, r. t., 90–120 min	Loaded in the hydrogel, it may act as a crosslinker	Human keratinocytes cells (HaCat); *Staphylococcus aureus* (*S. aureus*), *Acinetobacter baumani* (*A. baumani*) and *Klebsiella pneumoniae carbapenemase* (KPC); 7 and 8 weeks old mice.	The conjugation of chitosan and collagen with NAC and ε-PL supports cell viability in vivo and in vitro, reduces inflammatory cells, and induces collagenous expression of MMP-1.	
[31]	Chitosan modified with NAC (CS-NAC), genapping (GP), AgNO_3_	292.32 ± 5.98 μmol/g	Chemical r. t., 3 h	Modifying agent, it is crosslinked to silver ions	*E. coli* and *S. aureus*; L929 cells.	Increased cell proliferation and good cytocompatibility. Antibacterial effects against *E. coli* and *S. aureus* due to Ag-S coordination.	
[45]	Acrylic acid (AA), gelatin (Gel), CS-NAC, poly(vinyl butyral) (PVB), Gelatin Methacryloyl (GelMA), α-ketoglutaric acid	N.I.	UV-vis, r. t., 20 min	Modifying agent	Epithelial cells (IOSE80 cells) and rat cardiomyocytes; Sprague-Dawley male rats.	Good adhesion, capacity to adhere strongly to the tissue surface, and easy detachment by the application of a solution.	
[46]	Chitosan modified with NAC and NO group (CS-SNO); Hyaluronic acid modified with ethane diamine (HA-NH2); Polyethylene glycol modified with 4-formylbenzoic acid (APG)	12.85%	Chemical, r. t., 20–50 s	Modifying agent	*E. coli* and *S. epidermidis*; NIH 3 T3, HFF-1 and bEnd.3 cells.	Antibacterial activity against *E. coli.* Promotes cell growth and wound closure at lower concentrations of CS-NO, pH-responsive gelation, and self-healing.	
[10]	Aldehyde-modified hyaluronic acid (SA-HA) and thioketal linker with amino end groups (TK-NH2), NAC	N.I.	Chemical, r. t., <10 min	Loaded into the hydrogel	NIH 3 T3 cells; Red blood cells from freshly collected mouse blood; *S. aureus* and *E. coli*; ICR mice.	Significant ROS scavenging effect is higher than the components separately. Antibacterial effect against *E. coli* and *S. aureus*, good cell viability, and biocompatibility in vitro, with a hemolysis rate lower than 3%. Biocompatibility in vivo. Fast healing in burn wounds, with the highest closure rate, hair growth, attenuated response, and collagen deposition. Self-healing and ROS-responsive.	
[30]	CS-NAC, dopamine-modified Poly (lactic-co-glycolic acid) (PLGA) fibers (PLGA-PDA), β-glycerol phosphate (β-GP)	N.I,	Chemical, 37 °C, 24 h	Modifying agent.	Sprague-Dawley (SD) rat bone marrow mesenchymal stem cells (BMSCs, in vitro); 6 week olds (ectopic implantation), and 4- week-old SD rats (in situ transplantation).	Good cell viability and in vitro biocompatibility. Cell migration was higher when fibers were added. Slightly higher proteoglycan expression was observed than that in the control group. Hemolysis rate below 5%, indicating good blood compatibility. In vivo, fibers play a major role in tissue regeneration. Thermosensitive gelation.	
[47]	NAC-modified gelatin (Gel-SH), methacrylate silk fibroin (SF), (-)-epi-gallocatechin gallate (EGCG)–copper ionic–κ-carrageenan (κ-CA) nanoparticles (EG−Cu−CA NPs), lithium phenyl-2,4,6-trimethylbenzoylphosphinate (LAP)	185 mmol/g	UV-vis, 30 s	Modifying agent	*E. coli*, *S. aureus*, and Methicillin-resistant *S. aureus* (MRSA). NIH 3T3 and human umbilical vein endothelial cells (HUVECs); SD rats.	The ROS-scavenging effect was observed even for hydrogel NPs, hemocompatibility, good healing properties induced by the hydrogel matrix alone, and accelerated by NPs. Inflammatory response was observed in the group containing only the matrix hydrogel.	
[25]	CS-NAC, benzaldehyde-terminated polyethylene glycol (POSS-PEG-CHO), molybdenum disulfide nanosheets (MoS_2_ Ns)	0.32	Chemical, 37 °C, 5 min	Modifying agent	HUVECs; *S. aureus* and *E. coli*; hemorrhagic mouse liver (ex vivo); Diabetic mouse infected with *S. aureus*.	Fast gelation, self-healing, injectability, biocompatibility, photothermal capacity, antioxidant activity, hemostatic capacity, and in vivo healing capacity. The hydrogel showed excellent antibacterial effect without NIR laser radiation, approximately 96% for both cultures. The effect increased to 100% with NIR laser radiation. The hydrogel and raw materials were virtually free of cytotoxicity, with or without NIR irradiation.	
[26]	Col, graphene oxide (GO), NAC, EDC, n-hydroxy succinimide system (NHS)	N.I.	Chemical, r. t., 24 h	Modifying agent	NIH 3T3 and Hakata; 6 weeks old SD male rats.	Diabetic wound healing and angiogenesis accelerate extracellular matrix synthesis and antioxidant activity.	
[34]	Col, EDC and NHS, NAC and polyamide nanofibers (PA)	N.I.	Chemical, r. t., 24 h	Modifying agent	NIH 3T3 cells; 8–10 weeks old male SD rats.	Biocompatible, non-cytotoxic, sustained NAC release, and good wound healing. The NAC-treated groups showed a higher percentage of wound closure, approximately Wound healing 86.17% on day 14. The scaffolds showed a continuous epidermis, high collagen deposition, and regularly arranged new collagen mass.	
[32]	Carboxymethyl chitosan (CMCS), oxidized hyaluronic acid (OHA), ε-poly-L-lysine-S-Aroylthiooximes (ε-PL-SATO), NAC	N.A.	Chemical, r. t., <30 s	Loaded in the hydrogel	L929 cells; red blood cells from fresh blood of SD rats (hemolysis) *E. coli* and *S. aureus;* SD male rats	fast gelation time, needle injectability, self-healing, H_2_S release, NAC-dependent, antibacterial activity, biocompatibility, and good wound healing. Hemolysis rate is less than 4%, colorless and transparent. Wound healing was almost complete (99%) after 14 days.	
[43]	Collagen sponges (CollaPlug) and Collagen membrane (Bio-Gide), NAC	N.A.	N.I.	Loaded in the hydrogel	Oral mucosal cells from Sprague-Dawley rats (in vitro)	Oral soft-tissue growth. The viability of oral mucosal cells increased with NAC treatment; however, hyperproliferation and collagen deposition were abrogated. Collagen-related gene transcription was downregulated with increasing NAC concentration. The addition of NAC reduced the rate of cell proliferation in a dose-dependent manner.	
[44]	CS-NAC, maleic acid modified dextran (Dex-Ma), Basic fibroblast growth factor (bFGF)	N.I.	Chemical, 37 °C, <1 min	Modifying agent	NIH3T3 cells; Male SD rats.	Healing properties, ability to carry cellular growth factors, drug loading, and release. After 9 days of treatment, the group treated with the bFGF-loaded hydrogel showed fewer inflammatory cells than the other three groups, and the formation of new blood vessels and fibroblast proliferation were observed in the granulation tissue of the hydrogel-treated group.	
[29]	Carbopol^®^ Ultrez NF 10, NAC, lysine (LYS)	N.A.	N.I.	Loaded into the hydrogel	HaCaT and THP-1 cells.	Biocompatible, drug release, and self-healing. The hydrogel formulation with active ingredients at 20 mM and 100 mM did not show any significant irritant activity, with tissue viability higher than 94% compared to the control.	
[36]	Col type I, NAC, GO	N.I.	Chemical	Modifying agent	NIH 3T3 cells; 8–10 weeks old SD male rats.	Biocompatibility, wound healing, drug release. AC reduced the cytotoxicity of GO, downregulated the levels of ROS induced by GO, and promoted the fastest cell migration when aligned with GO.	
[93]	ALG, NAC, CaCl_2_	N.I.	Physical, 25 °C	Loaded into the hydrogel	MC3T3 cells.	Drug release, wound healing, and re-epithelialization. Reduction in cell death associated with extrusion process. NAC improved cell viability and reduced apoptotic and inflammatory expression.	
[87]	NaHA modified with cystamine dihydochloride, PDA-PEG-NAC, PDA-PEG-bFDF	72.1 μmol/g	Chemical, 5 min	Modifying agent	bFDF; 10–12 weeks old C57BL/6 diabetic mice (blood glucose levels exceeding 16.7 mmoL/mL).	Drug and cell release, anti-inflammatory activity, biocompatibility, wound closure.	
[37]	CS-NAC, silver nanowire (AgNW), glass substrate	N.I.	Chemical	Modifying agent, crosslinked	*E. coli* and *S. aureus* by the zone of inhibition; Human hepatic cell line L02; 16 months old pregnant rabbits.	Antibacterial effect, biocompatible, and wound healing, low cytotoxicity	
[48]	Catechol-conjugated chitosan modified with thiol group (CSDS), Sodium periodate (NaIO_4_)	75.21 ± 5.2 μmol/g	Chemical, 30 s	Modifying agent, crosslinked	L929 cells.	Adhesive and biocompatible.	
[40]	CS-NAC, maleimide group modified ε-polylysine (EPLM)	151.8 μmol/g	Chemical, r. t., 15 ± 3 s	Modifying agent, crosslinked	L929 cells. 6 weeks old SD male rats (hemostatic ability in vivo).	Non-cytotoxicity in vitro, rapid gelation, and excellent adhesion. The blood loss from the liver was reduced from 106.7 mg (untreated liver) to 28.3 mg due to the good adhesiveness of the hydrogel.	
[33]	Col, NAC, Poly (ε-caprolactone) (PCL) fibers	N.I.	Chemical, r. t., 8 h	Modifying agent	NIH 3 T3 cells; 8–10 weeks old SD male rats.	It is biocompatible and highly porous, promoting cell proliferation, wound healing, angiogenesis, and sustained drug release.	
[27]	Poly (ethylene adipate) diol ((PEA) diol), ε-Caprolactone, Rac-Lactide and Poly(ethylene glycol) copolymer (CL-LA-PEG200), CS, 1,6-diisocyanatohexane (hexamethylene diisocyanate (HDI), NAC beads	N.A.	Physical, r. t., 24 h	Loaded in the hydrogel	10 weeks old diabetic male mice (glucose serum concentration was 483 ± 191.9 mg/dL).	Fast wound closure, re-epithelialization, sustained drug release, and biocompatibility. The 5% NAC hydrogels showed better results for wound closure than those treated with 10% and 20% NAC.	
[35]	NAC, silver salts and asiatic acid-poly(aniline)-quercetin (AA-PANi-Q) nanomaterial	N.A.	Chemical (coordination)	Complexation agent	*E. coli* and *S. aureus*; L929 cells.	Non-cytotoxicity, antibacterial effect, biocompatibility, controlled drug delivery, photothermal responsiveness, and structural stability. A modest reduction in bacterial colonies was observed with NIR irradiation. The group containing only NAC and silver displayed a more effective bactericidal outcome against *S. aureus* than against *E. coli*. Overall, a synergistic effect was observed between the formulation and photothermal activation.	
[39]	Sodium alginate (SA), guar gum (GG), NAC	N.A.	Physical	Loaded into the hydrogel	*S. aureus*, *E. coli*, *P. aeruginosa* and *Candida albicans* (*C. albican*).	Cell proliferation, antimicrobial, antibiofilm, and antioxidant activities and low cytotoxicity. Free NAC inhibited *E. coli* biofilm formation by up to 75.80%, whereas the hydrogel (2% *v*/*v* SA, 1% *v*/*v* GG, 05 mg/mL NAC) inhibited *S. aureus* biofilm formation by 28.29% even at low doses (12.5 μg/mL).	
[38]	PCL, (GO), NAC modified chitosan-co-polycaprolactone (NAC-CS-PCL)	N.I.	Chemical, 24 h	Modifying agent	Human dermal fibroblasts (HDFs); SD male rats.	Angiogenesis, collagen deposition, and biocompatibility. NAC induced slight cell proliferation. In vivo, only the NAC-treated group displayed newly formed blood vessels.	
[41]	Polyimidazom containing maleimide terminal groups (PIM(Cn)-Mal), poly(ethylene glycol) tetra thiol (PEG-4SH), poly(ethylene glycol) tetra maleimide (PEG-4mal), thiol-functionalized alginate (Alg-SH), PEG-2Mal-2NAC	N.I.	Chemical, 25 °C, 5 min	Modifying agent	MRSA USA300, CR-AB, PAO1 or CR-PA; N3T3 and HDFs; 8 weeks old male C57BL/6 mice.	Accelerating the healing of diabetic wounds infected by biofilms. Infected wounds were closed in 12 days in vivo.	
[50]	Vancomicin, CS-NAC, Dex-Ma	N.I.	Chemical, 37 °C, <1 min	Modifying agent	NIH3T3 cells; *S. aureus* and *E. coli*.	Biocompatible, drug administration, antimicrobial The hydrogel group without vancomycin showed antibacterial activity against Gram-negative bacteria (*E. coli*).	
[88]	Polyethylene glycol diacrylate (PEGMA), tannic acid (TA), NAC, and silver nitrate	N.A.	Chemical	Complexation agent	*E. coli* and *S.aureus*; NIH3T3 cells.	Injectable, antibacterial activity, antibiofilm activity, sustained drug release for 8 h, scavenger activity, non-cytotoxic.	
[89]	Polymethylmethacrylate (PMMA), marine collagen (MCol), NAC	N.I.	Physical, 25 °C	Loaded into the hydrogel	Human normal fibroblast cell line (BJ1); Strains of *Sepidermidis*, *S. aureus*, *Bacillus cereus*, *Salmonella paratyphi*, *E. coli*, *Klebsiella pneumoniae*, *Candida glabrata*, *Candida albicans*, and *Candida parapsilosis*.	Biocompatibility, antioxidant, and antimicrobial properties.	
