# Supplementary material for: N-Acetylcysteine Applied to Hydrogels: A Comprehensive Systematic Review
Source: Gels. 2026 Aug 21;12(8):751. doi: 10.3390/gels12080751 (PMC13512807; doi:10.3390/gels12080751)
Supplement: Supplementary file 1 [file gels-12-00751-s001.zip › gels-4440107-supplementary-File S1.pdf]

PRISMA 2020 Checklist

Section and Topic	Item #	Checklist item	Location where item is reported
**TITLE**			
Title	1	Identify the report as a systematic review.	p. 1, title, lines 2–3.
**ABSTRACT**			
Abstract	2	See the PRISMA 2020 for Abstracts checklist.	p. 2, Abstract, approximately lines 16–54 (partial PRISMA abstract reporting).
**INTRODUCTION**			
Rationale	3	Describe the rationale for the review in the context of existing knowledge.	pp. 2–3, Introduction, lines 49–104
Objectives	4	Provide an explicit statement of the objective(s) or question(s) the review addresses.	pp. 3 lines 105-107.
**METHODS**			
Eligibility criteria	5	Specify the inclusion and exclusion criteria for the review and how studies were grouped for the syntheses.	p. 3, lines 114-117; p. 3, lines 119-130. Study grouping for synthesis described in Figure 5.
Information sources	6	Specify all databases, registers, websites, organisations, reference lists and other sources searched or consulted to identify studies. Specify the date when each source was last searched or consulted.	p. 3, lines 109-110, 118. Scopus, SciFinder, PubMed and Web of Science are named; last-search dates line 115.
Search strategy	7	Present the full search strategies for all databases, registers and websites, including any filters and limits used.	p. 3, lines 110-121; p. 3, lines 109-119 for SciFinder. The specific search string is provided.
Selection process	8	Specify the methods used to decide whether a study met the inclusion criteria of the review, including how many reviewers screened each record and each report retrieved, whether they worked independently, and if applicable, details of automation tools used in the process.	p. 3, lines 119-130; and Figure 1. Zotero and automated exclusions are mentioned; 02 reviewers screened (E.S.Junckes and P. E. Munzlinger) as described in the “Author Academic Background Form”. Independence and conflict resolution are not reported.
Data collection process	9	Specify the methods used to collect data from reports, including how many reviewers collected data from each report, whether they worked independently, any processes for obtaining or confirming data from study investigators, and if applicable, details of automation tools used in the process.	Not reported in the current draft. 02 reviewer screened (E.S.Junckes and P. E. Munzlinger) as described in the “Author Academic Background Form”. A third person (M.M.M) assisted in case of uncertainty, p.3 line 124-128.
Data items	10a	List and define all outcomes for which data were sought. Specify whether all results that were compatible with each outcome domain in each study were sought (e.g. for all measures, time points, analyses), and if not, the methods used to decide which results to collect.	Partly indicated in the Abstract (p. 1) and objective (p. 2, lines 66–68); Figure 5 (p. 6). and Table 1-3 (pp.11-14, pp. 22-23 and pp. 25-28) and Table A1 (pp. 38-52) resume the main outcomes considered.
	10b	List and define all other variables for which data were sought (e.g. participant and intervention characteristics, funding sources). Describe any assumptions made about any missing or unclear information.	Table 1 (pp. 11-14) reports substance released, experimental conditions of release assay, swelling degree, cumulative release behavior, and intended application of NAC. Table 2 (pp. 22-23) reports main antimicrobial and antibiofilm effects. Table 3 (pp. 25-28) reports main in vivo assays, animal model, assay description, hydrogel and main results.
Study risk of bias assessment	11	Specify the methods used to assess risk of bias in the included studies, including details of the tool(s) used, how many reviewers assessed each study and whether they worked independently, and if applicable, details of automation tools used in the process.	Not reported; no study-level risk-of-bias assessment method or tool is described.
Effect measures	12	Specify for each outcome the effect measure(s) (e.g. risk ratio, mean difference) used in the synthesis or presentation of results.	N/A — no pooled quantitative synthesis or meta-analysis was performed; no prespecified effect measure is reported.
Synthesis methods	13a	Describe the processes used to decide which studies were eligible for each synthesis (e.g. tabulating the study intervention characteristics and comparing against the planned groups for each synthesis (item #5)).	Not reported as a method. The narrative results are grouped thematically in Sections 3-6 (pp. 5-31) and Table 1A (pp. 38-52).
	13b	Describe any methods required to prepare the data for presentation or synthesis, such as handling of missing summary statistics, or data conversions.	N/A - no quantitative data analysis, conversion, imputation or preparation method was done.
	13c	Describe any methods used to tabulate or visually display results of individual studies and syntheses.	Results are displayed in Figures 2–13 (pp. 4-8, 15-17, 22, 24, 28 and 31) and Table 1-3 (pp. 11-14, 22-23, 25-28) and Table A1 (pp. 38-52)
	13d	Describe any methods used to synthesize results and provide a rationale for the choice(s). If meta-analysis was performed, describe the model(s), method(s) to identify the presence and extent of statistical heterogeneity, and software package(s) used.	No meta-analysis was done. The results were synthesized in Table 1. Also, to synthesize results a thematic narrative is presented in Sections 2–6 (pp. 4–31).

PRISMA 2020 Checklist

Section and Topic	Item #	Checklist item	Location where item is reported
	13e	Describe any methods used to explore possible causes of heterogeneity among study results (e.g. subgroup analysis, meta-regression).	N/A — no formal subgroup analysis, meta-regression or other investigation of heterogeneity was conducted.
	13f	Describe any sensitivity analyses conducted to assess robustness of the synthesized results.	N/A — no sensitivity analysis was conducted.
Reporting bias assessment	14	Describe any methods used to assess risk of bias due to missing results in a synthesis (arising from reporting biases).	Not reported; reporting-bias or missing-results bias was not assessed.
Certainty assessment	15	Describe any methods used to assess certainty (or confidence) in the body of evidence for an outcome.	N/A — certainty/confidence in the body of evidence was not assessed.
**RESULTS**			
Study selection	16a	Describe the results of the search and selection process, from the number of records identified in the search to the number of studies included in the review, ideally using a flow diagram.	p. 3 and 4, Figure 2 and lines 115-133: 614 records identified, 216 duplicates and 188 automated exclusions removed, 398 screened, 92 full texts assessed and 73 studies included.
	16b	Cite studies that might appear to meet the inclusion criteria, but which were excluded, and explain why they were excluded.	p. 3-4, lines 116-130. One report was not retrieved and 19 full texts were excluded for not reporting hydrogel systems; excluded reports are not individually cited.
Study characteristics	17	Cite each included study and present its characteristics.	Table A1, pp. 38-52, and narrative Sections 2–6 (pp. 4–31); included studies are cited and their main characteristics are reported.
Risk of bias in studies	18	Present assessments of risk of bias for each included study.	Not reported; no risk-of-bias results are presented for included studies.
Results of individual studies	19	For all outcomes, present, for each study: (a) summary statistics for each group (where appropriate) and (b) an effect estimate and its precision (e.g. confidence/credible interval), ideally using structured tables or plots.	Table A1, pp. 38-52, and Sections 4–6, pp. 9-31. Study-level outcomes and selected numerical findings are reported; effect estimates and precision are not consistently available.
Results of syntheses	20a	For each synthesis, briefly summarise the characteristics and risk of bias among contributing studies.	Narrative thematic syntheses in Sections 2–6 pp. 4–31; study characteristics in Table A1, pp. 38-52. Risk of bias is not summarized.
	20b	Present results of all statistical syntheses conducted. If meta-analysis was done, present for each the summary estimate and its precision (e.g. confidence/credible interval) and measures of statistical heterogeneity. If comparing groups, describe the direction of the effect.	N/A — no statistical synthesis or meta-analysis was performed.
	20c	Present results of all investigations of possible causes of heterogeneity among study results.	N/A — no formal investigation of causes of heterogeneity was conducted.
	20d	Present results of all sensitivity analyses conducted to assess the robustness of the synthesized results.	N/A — no sensitivity analysis was conducted.
Reporting biases	21	Present assessments of risk of bias due to missing results (arising from reporting biases) for each synthesis assessed.	Not reported; risk of bias due to missing results was not assessed.
Certainty of evidence	22	Present assessments of certainty (or confidence) in the body of evidence for each outcome assessed.	Not reported; certainty of evidence was not assessed.
**DISCUSSION**			
Discussion	23a	Provide a general interpretation of the results in the context of other evidence.	Section 7, Conclusion, pp. 31-32, lines 488-554
	23b	Discuss any limitations of the evidence included in the review.	Abstract, p. 1, lines 34–36; Conclusion, pp. 31-32, lines 488-554. Heterogeneity and limitations of the evidence are discussed.
	23c	Discuss any limitations of the review processes used.	Not reported; limitations of the review process itself are not explicitly discussed.
	23d	Discuss implications of the results for practice, policy, and future research.	Abstract, p. 1, lines 36–41; Conclusion, 31-32, lines 488-554, including recommendations for characterization, release studies and preclinical validation.
**OTHER INFORMATION**			
Registration and protocol	24a	Provide registration information for the review, including register name and registration number, or state that the review was not registered.	Not reported. No register name or registration number is provided; the manuscript should state that the review was not registered if applicable.
	24b	Indicate where the review protocol can be accessed, or state that a protocol was not prepared.	Not reported. No protocol or protocol-access statement was prepared.

PRISMA 2020 Checklist

Section and Topic	Item #	Checklist item	Location where item is reported
	24c	Describe and explain any amendments to information provided at registration or in the protocol.	N/A — no registration or protocol is reported, so amendments cannot be described.
Support	25	Describe sources of financial or non-financial support for the review, and the role of the funders or sponsors in the review.	p.33, lines 562-567, Acknowledgments, approximately lines 569-578. CAPES, CNPq, FAPESC (2025TR001425), PDSE, UDESC and CNR are acknowledged; funder roles are not stated.
Competing interests	26	Declare any competing interests of review authors.	p. 33, Conflicts of Interest, line 579: authors declare no conflicts of interest.
Availability of data, code and other materials	27	Report which of the following are publicly available and where they can be found: template data collection forms; data extracted from included studies; data used for all analyses; analytic code; any other materials used in the review.	p. 33, Data Availability Statement, approximately lines 568. The statement refers readers to the cited publications; extracted datasets/forms/code are not reported as publicly available.

From: Page MJ, McKenzie JE, Bossuyt PM, Boutron I, Hoffmann TC, Mulrow CD, et al. The PRISMA 2020 statement: an updated guideline for reporting systematic reviews. BMJ 2021;372:n71. doi: 10.1136/bmj.n71. This work is licensed under CC BY 4.0. To view a copy of this license, visit https://creativecommons.org/licenses/by/4.0/
